# Synthetic augmentation in ACL reconstruction may reduce re‐rupture rates and increase return‐to‐sport rates: A systematic review and meta‐analysis

**DOI:** 10.1002/ksa.12680

**Published:** 2025-04-18

**Authors:** Yufei Jiang, Seyran Naghdi, Nick Smith, Toby Smith, Andrew Metcalfe, Hema Mistry

**Affiliations:** ^1^ Warwick Clinical Trials Unit, Warwick Medical School University of Warwick Coventry UK; ^2^ University Hospitals Coventry and Warwickshire NHS Trust Coventry UK

**Keywords:** anterior cruciate ligament, efficiency of ACL synthetic augmentation, knee, review of synthetic ACL reconstruction

## Abstract

**Purpose:**

Synthetic augmentation (SA) in anterior cruciate ligament reconstruction (ACLR) aims to enhance graft durability, but its benefits remain unclear. To evaluate whether SA in ACLR improves return‐to‐sport (RTS) rates, reduces graft failure, enhances patient‐reported outcomes (PROs) and varies in effectiveness across materials and techniques.

**Methods:**

A systematic search of five databases was conducted until February 2025. Comparative studies were pooled using Hedges' random‐effects meta‐analysis with subgroup analysis based on materials and publication year. Non‐comparative studies were analysed narratively. Risk of bias was assessed using the Risk of Bias in Non‐randomised Studies of Interventions and the Cochrane risk‐of‐bias tools for randomised studies. Grades of Recommendation, Assessment, Development and Evaluation (GRADE) approach was used to assess the certainty of evidence.

**Results:**

Forty‐seven studies were included (*n* = 4289): 7 randomised and 40 non‐randomised studies (21 comparative; 19 non‐comparative). SA systems included InternalBrace (FiberTape, 16 studies), Ligament Augmentation and Reconstruction System (polyester, 5), Ligament Augmentation Device (polyethylene, 18) and other materials (8). GRADE assessment showed moderate‐certainty evidence for improved mid‐term RTS rate from eight studies (odds ratio [OR]: 1.58; 95% confidence interval (CI): 1.12–2.22; *N* = 716; *I* ^2^ = 0%; *p* = 0.01). Internal brace showed a reduction in re‐rupture rates in the long‐term (OR: 0.17, 95% CI: 0.04–0.64; *N* = 218; *I* ^2^ = 0%; *p* = 0.01); however, pooled analysis of all techniques showed no statistically significant difference. Contemporary studies showed a better return to sport rates with SA. PROs showed no clinically meaningful differences. Non‐comparative studies showed low graft failure rates (<8.7% for InternalBrace; <16.4% for other SA), high RTS rates (>90% for InternalBrace; >56.7% for other SA) and satisfactory PROs.

**Conclusions:**

SA, particularly InternalBrace, may improve RTS rates and reduce re‐rupture risk, though PROs remain inconclusive. Findings are limited by a moderate‐to‐serious risk of bias, emphasising the need for high‐quality research.

**Level of Evidence:**

Level III.

**Abbreviations**
ACL: anterior cruciate ligament
ACLR: anterior cruciate ligament reconstruction
BMI: body mass index
CI: confidence interval
IKDC: International knee documentation committee
KOOS: Knee Injury and Osteoarthritis Outcome Score
MD: mean difference
OR: odds ratio
PET: polyethylene terephthalate
PRISMA: Preferred Reporting Items for Systematic Reviews and Meta‐Analyses
RTS: return to sport
SA: synthetic augmentation
SD: standard deviation

## INTRODUCTION

Anterior cruciate ligament (ACL) tears have an incidence rate of 1 in 50 in male athletes and 1 in 29 in female athletes [43], particularly those undertaking pivoting sports [28]. ACL reconstruction (ACLR) is the ‘gold standard’ for treating ACL tears, especially in athletes [6, 58]. However, only 55% of patients successfully return to their pre‐injury sport level following surgery [1]. Graft failure rates vary between 15% and 40% in high‐risk individuals, emphasising the need for improved surgical techniques to enhance graft durability and functional recovery [27, 31, 61].

Various intra‐articular and extra‐articular reinforcement techniques, including synthetic augmentation (SA), have been developed [18, 27]. SA is designed to enhance graft strength, reduce elongation and improve biomechanical stability, particularly in high‐risk populations [37]. By increasing initial reconstruction strength, SA also helps protect the autograft prior to graft remodelling and maturation [49]. Early‐generation synthetic ligaments, including carbon fibre and Dacron, demonstrated poor biological integration, leading to high rates of graft rupture and joint inflammation [37]. However, advancements in biomaterials have improved durability and biocompatibility, prompting renewed interest in SA [70]. Biomechanical studies suggest that suture tape augmentation can improve graft strength and reduce elongation under cyclical loading [39]. This may then lead to a stronger and less lax graft, which could result in better return‐to‐sport (RTS) rates and reduced re‐rupture rates [71]. Various SA types have been explored, including non‐resorbable materials such as polyethylene terephthalate (PET) and polyurethane, resorbable synthetic scaffolds and polypropylene meshes used to wrap the graft for additional support.

Historically, synthetic substitutes (e.g., Ligament Advanced Reinforcement System [LARS], Kennedy Ligament Augmentation Device [LAD]) were intended as primary ACL graft alternatives but exhibited high failure rates due to poor biological integration and mechanical wear. In contrast, synthetic augments are designed to reinforce autografts rather than replace them [37]. As a result, their failure mechanisms also differ: traditional augments primarily failed due to mechanical fatigue, whereas substitutes were more prone to synovitis and poor tissue incorporation [19, 37]. Understanding these differences is crucial for evaluating modern SA strategies [37].

Previous systematic reviews (2023 and 2024) have examined the outcomes of ACLR with only suture (tape) augmentation and internal brace compared to ACLR alone [18, 25, 77]. These reviews indicated that while SA shows potential, there is currently insufficient evidence, or low confidence in the evidence to support its superiority over traditional ACLR [18, 25, 77]. However, these reviews were limited to specific materials and comparator studies. Our study provides new and novel findings, expanding on these previous systematic reviews by quantitatively assessing the effectiveness of all types of SA in ACL injury management. Including non‐comparator studies broadens the assessment of augmentation techniques and long‐term outcomes. Despite a lower level of evidence, they offer real‐world insights that complement higher‐quality studies [10, 11].

## METHODS

This review is reported following Preferred Reporting Items for Systematic Reviews and Meta‐Analyses (PRISMA) guidelines [51] and the Cochrane Handbook for Systematic Reviews of Interventions [9]. The protocol is registered in the PROSPERO database (reference: CRD42024516626).

### Inclusion criteria

Inclusion criteria were clinical effectiveness studies on ACLR with SA on grafts for individuals with ACL injuries. Studies of all levels of evidence were included. Exclusion criteria included ACL repair, co‐interventions, deliberate selection of undersized grafts, non‐human studies, studies unrelated to the knee and biomechanical/cadaveric studies. If a study compared ACLR with SA versus other interventions (e.g., ACLR using synthetic graft alone), only the SA ACLR group were extracted and analysed if separate outcome data were available. This approach prevents confounding and ensures consistency in evaluating SA effectiveness [9].

### Search strategy and selection criteria

A search of five published and unpublished literature databases, including MEDLINE, Embase, Web of Science, Global Index Medicus and Clinicaltrials.gov, was conducted up to 17 February 2025. The search strategies are presented in Supporting Information S2: Appendix 1. One reviewer (SN) performed the search, which was verified by a second information specialist (AB). Including ClinicalTrials.gov helped identify ongoing or unpublished trials, but none met the inclusion criteria [5, 29].

Title and abstract screening according to population, intervention, comparator and outcomes criteria were conducted by two reviewers (SN and YJ) independently. The same two reviewers (SN and YJ) reviewed the full texts of the studies according to the prespecified inclusion/exclusion criteria. In cases of disagreement, a third reviewer (TS and NS) adjudicated.

### Data extraction

Data extraction was performed by one reviewer (YJ) and independently reviewed by a second (SN). Data on the title, author, year of publication, study design, level of evidence, number of participants, baseline characteristics, details of the intervention and comparator, study follow‐up period, inclusion and exclusion criteria, and outcomes of interest were extracted using a prespecified spreadsheet. Means and standard deviations (SDs), mean differences (MDs) and 95% confidence intervals (CIs) for continuous outcomes and proportions for binary outcomes were extracted. When SDs were not provided, they were calculated using Cochrane's recommended methods for meta‐analysis where possible [33].

Studies were included if they reported at least one of the predefined outcomes of interest. Not all studies provided data for every outcome.

### Outcome measures

The primary outcomes were:

1. Failure rate, re‐rupture or revisions
2. RTS activities, return‐to‐work activities rate, and ACL RTS after injury (ACL‐RSI) survey score [13, 22].

The secondary outcomes were patient‐reported outcomes (PROs) including a Knee Injury and Osteoarthritis Outcome Score (KOOS), Lysholm score, Tegner activity level and International Knee Documentation Committee (IKDC) score.

### Follow‐up timepoint categorisation

To ensure consistency in reporting, outcomes were categorised based on follow‐up periods: short‐term (<12 months), mid‐term (12–48 months) and long‐term (>48 months).

### Assessment of risk of bias and certainty in evidence

Two reviewers (SN and YJ) independently performed the quality assessment and certainty of evidence, and any discrepancies were discussed until a consensus was reached. Bias was assessed using the Risk Of Bias In Non‐randomised Studies – of Interventions (ROBINS‐I) tool [66] and the Revised Cochrane Risk of Bias tool for randomised trials (RoB 2) [67] in Excel. The certainty of evidence for the meta‐analysis estimates was assessed using the Grades of Recommendation, Assessment, Development and Evaluation (GRADE) approach [54].

### Data analysis

The results of the non‐comparative studies were reported narratively in subgroups by intervention type, follow‐up period and outcome, and key findings were summarised to complement the comparative studies where applicable. For comparative studies, when study homogeneity was deemed appropriate based on study design, population, and intervention, a meta‐analysis using Hedges' random effects model [32] was conducted to compare outcomes between patients undergoing ACLR with SA and those undergoing ACLR alone. Forest plots were created to display effect sizes and 95% CIs. Statistical heterogeneity was assessed with the *I* ^2^ statistic, where values above 50% indicated significant heterogeneity [34]. Publication bias was evaluated using funnel plots and Egger's test. Additionally, subgroup analyses were performed for different types of augmentation materials where possible. Also, a sensitivity analysis based on the year of publication has been conducted to compare recent studies published within the past 10 years versus the older ones. All analyses were performed with STATA 18 [64]. Results were considered statistically significant at *p* < 0.05.

## RESULTS

### Study selection

**Figure 1:**
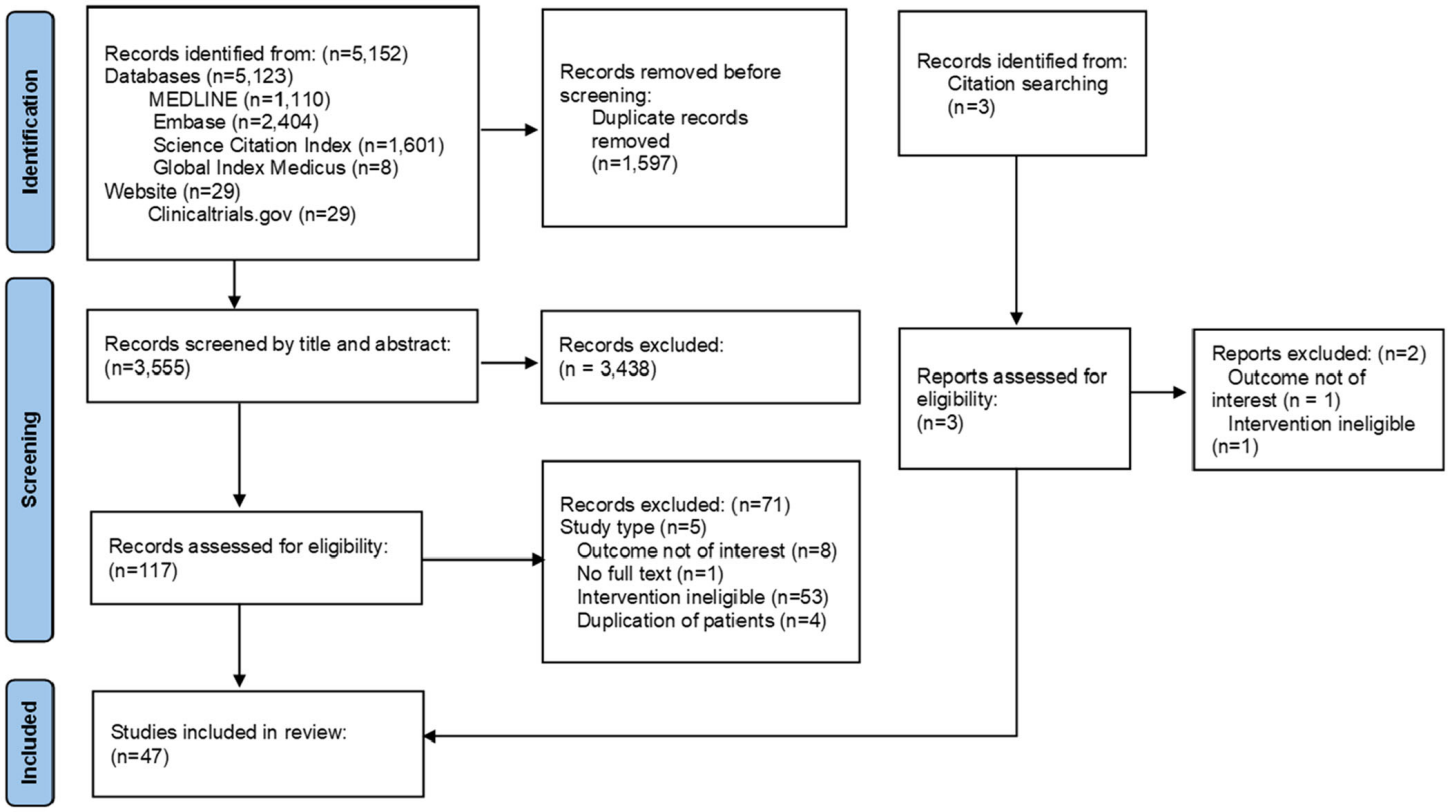
PRISMA flow diagram.

The search strategies yielded 3555 records after the removal of duplicates, and we identified three more studies by citation searching. We excluded 3438 citations after the title and abstract sifting. One hundred twenty records were obtained for full‐text screening (Supporting Information S2: Appendix 2). Forty‐seven studies [2, 3, 4, 8, 12, 13, 14, 15, 16, 17, 20, 21, 22, 23, 24, 26, 30, 35, 36, 38, 40, 41, 42, 44, 45, 46, 47, 48, 50, 52, 53, 55, 56, 57, 59, 60, 62, 63, 65, 68, 69, 71, 72, 73, 74, 75, 76] met the eligibility criteria and were included in the analysis (Figure 1).

### Study characteristics

Twenty‐one [3, 4, 8, 14, 16, 17, 21, 36, 40, 42, 50, 52, 55, 56, 59, 60, 65, 71, 72, 74, 76] were non‐randomised comparative studies (Evidence level: 2 or 3) and seven [30, 41, 44, 46, 47, 53, 73] were randomised prospective comparative studies (Evidence level: 1). Fifteen included studies [2, 13, 15, 20, 21, 22, 24, 26, 38, 48, 57, 63, 68, 69, 75] were non‐comparative studies (Evidence level: 3 or 4). In addition, four studies [12, 35, 45, 62], which were comparative but did not compare SA ACLR with traditional ACLR, were viewed as non‐comparative studies; only outcome data of group(s) of interest were included in the analysis. In total, 4289 patients were included; 2822 underwent ACLR with SA; 1467 underwent ACLR without SA (comparator). Mean patient age ranged from 14.9 [36] to 37.2 [62] years. In one study [60], patients were followed for a short time only. Mid‐term outcomes were reported in 35 studies [2, 3, 4, 8, 12, 13, 15, 17, 20, 21, 22, 24, 26, 30, 36, 38, 41, 42, 44, 47, 48, 50, 52, 53, 56, 59, 62, 63, 68, 71, 72, 73, 74, 76]. Fourteen studies [14, 16, 17, 23, 35, 40, 45, 46, 53, 55, 57, 65, 69, 75] reported long‐term outcomes. Percentage of females ranged from 0% [69] to 68% [15] and the mean BMI ranged from 22.4 [44] to 28.5 [42] (Supporting Information S1: Table S1).

### Surgical technique

Surgical details of included studies [2, 3, 4, 8, 12, 13, 14, 15, 16, 17, 20, 21, 22, 23, 24, 26, 30, 35, 36, 38, 40, 41, 42, 44, 45, 46, 47, 48, 50, 52, 53, 55, 56, 57, 59, 60, 62, 63, 65, 68, 69, 71, 72, 73, 74, 75, 76] are reported in Supporting Information S2: Appendix 3. The anteromedial portal technique was reported to be used in 11 studies [13, 14, 16, 17, 21, 22, 41, 42, 52, 69, 74], the transtibial technique in 4 studies [8, 53, 68, 75], the all‐inside technique in 4 studies [20, 62, 63, 71], the outside‐in technique in 2 studies [36, 72] and (modified) over‐the‐top technique in 5 studies [12, 35, 46, 47, 55]. For femoral graft fixation, methods included suspensory devices, interference screws, staples, press‐fit positioning, and screws and washers. On the tibial side, the fixation methods varied similarly, with suspensory devices, interference screws and other techniques used across the studies. The InternalBrace technique, developed by Arthrex, was used in 16 studies [8, 13, 14, 15, 16, 17, 20, 22, 26, 36, 41, 52, 62, 63, 74, 75] for SA, primarily using FiberTape and SwiveLock anchors. Suture augmentation using FiberWire (Arthrex) was used in two studies [71, 72]. LARS was used in 5 studies [3, 21, 23, 24, 76], and LAD in 18 studies [2, 4, 12, 30, 35, 38, 40, 44, 45, 46, 47, 50, 55, 56, 57, 59, 65, 73]. Other types of SA, including woven polyester, polyethylene terephthalate, poly (urethane urea) devices and artificial ligament augmentation, were reported in six studies [42, 48, 53, 60, 68, 69].

### Quality and evidence‐certainty assessment

One randomised controlled trial [53] was judged as moderate risk of bias, primarily due to concerns about missing data, while the other six randomised studies [30, 41, 44, 46, 47, 73] were judged low risk of bias (Supporting Information S1: Figure S1) [67].

For the 39 non‐randomised studies [2, 3, 4, 8, 13, 14, 15, 16, 17, 20, 21, 22, 23, 24, 26, 35, 36, 38, 40, 42, 45, 48, 50, 52, 55, 56, 57, 59, 60, 62, 63, 65, 68, 69, 71, 72, 74, 75, 76] and 1 randomised study [12] where only SA ACLR group was included, evaluated using the ROBINS‐I tool [66]. Thirty‐nine studies [2, 3, 4, 8, 13, 14, 15, 16, 17, 20, 21, 22, 23, 24, 26, 35, 36, 38, 40, 42, 45, 48, 50, 52, 55, 56, 57, 59, 60, 62, 63, 65, 68, 69, 71, 72, 74, 76] showed a moderate risk of bias due to confounding while 1 [75] showed a serious risk. Most studies showed a low risk of bias in participant selection, except for four studies [17, 24, 36, 72] with moderate risk. Eighteen studies [13, 14, 15, 16, 21, 23, 24, 26, 36, 42, 45, 48, 56, 57, 63, 68, 69, 74] had a moderate risk of bias due to missing data, with one study [17] showing serious risk. Eleven studies [3, 4, 20, 36, 50, 56, 60, 65, 69, 74, 76] had a moderate bias in outcome measurement due to the potential lack of blinding and reliance on PROs, while the rest had low risk. Overall, two studies [17, 75] had a serious bias, while the others had a moderate risk (Supporting Information S1: Figure S2).

## META‐ANALYSIS AND NARRATIVE SYNTHESIS OF RESULTS

A summary of the findings is presented in Table 1.

- Graft failure
  Sixteen comparative studies [3, 8, 17, 30, 36, 41, 42, 47, 52, 53, 59, 71, 72, 73, 74, 76] reported graft failure rates in mid‐term follow‐up. Graft failure was mentioned directly in six studies [30, 36, 41, 42, 47, 52]. Re‐rupture/re‐tear is interpreted as graft failure in seven studies [3, 53, 71, 72, 73, 74, 76]. Revision ACLR is interpreted as graft failure in three studies [8, 17, 59]. The meta‐analysis, with moderate‐certainty evidence, revealed no significant difference in failure rates between the two groups overall (odds ratio [OR]: 0.70; 95% CI: 0.41–1.17, *N* = 1746; *I* ^2^ = 0%; *p* = 0.17; Supporting Information S1: Figure S3). Furthermore, the results of the meta‐analysis, including six studies [14, 16, 40, 46, 55, 65] reporting graft failure in the long‐term follow‐up, showed no significant difference between the two groups with moderate‐certainty evidence (OR: 0.55; 95% CI: 0.19–1.60, *N* = 479; *I* ^2^ = 11.18%; *p* = 0.27; Supporting Information S1: Figure S4). However, subgroup analysis showed a significant benefit for internal brace augmentation (OR: 0.17, 95% CI: 0.04–0.64, *N* = 218; *I* ^2^ = 0%; *p* = 0.01; Supporting Information S1: Figure S4).
  In addition, 13 non‐comparative studies [2, 12, 13, 15, 20, 21, 22, 24, 26, 38, 48, 62, 63] investigating ACLR with SA reported failure rate in mid‐term follow‐up. The graft failure rate ranged from 0.00% [15, 20, 63] to 8.7% [26] for the InternalBrace group and 0.00% [12, 48] to 10.81% [38] for the other SA. One study [75] reported a long‐term graft failure rate of 1.10% for patients undergoing ACLR augmented by InternalBrace. Four studies [35, 45, 57, 69] reported long‐term graft failure rates ranging from 1.87% [57] to 16.36% [69] for patients undergoing hybrid ACLR augmented by synthetic ligament or LAD.
- RTS rate
  Eight studies [3, 8, 36, 44, 50, 52, 56, 59] compared the RTS rates between SA and control groups in mid‐term follow‐up. RTS was defined as a return to pre‐injury sports activity at any level. The meta‐analysis indicated moderate‐certainty evidence that individuals who underwent SA had higher RTS rates compared to standard ACLR (OR: 1.58; 95% CI: 1.12–2.22, N = 716; *I* ^2^ = 0%; *p* = 0.01; Supporting Information S1: Figure S5). Subgroup analysis revealed that this effect was driven by the InternalBrace group (OR: 2.19; 95% CI: 1.18–4.04, *N* = 248; *I* ^2^ = 0%; *p* = 0.01; Supporting Information S1: Figure S5), while both the LARS and Kennedy LAD groups showed no differences in RTS rates between two groups. Also, moderate‐certainty evidence from five studies [14, 16, 23, 40, 55] showed no overall benefit of SA ACLR compared to traditional ACLR in terms of long‐term RTS (OR: 1.68; 95% CI: 0.70–4.08, *N* = 515; *I* ^2^ = 67.53%; *p* = 0.25; Supporting Information S1: Figure S6), while subgroup analysis showed a significant benefit of Kennedy LAD augmentation (OR: 6.01; 95% CI: 2.29–15.76, *N* = 167; *I* ^2^ = 0%; *p* < 0.01; Supporting Information S1: Figure S6).
  Additionally, five non‐comparative studies [15, 24, 38, 48, 69] reported on RTS rates. Except for one study on LARS‐augmented ACLR which reported a return‐to‐sport rate of 56.7% [24], all other studies reported relatively higher RTS rates for patients undergoing ACLR with an SA, ranging from 86.5% (semitendinosus and gracilis (STG) autograft augmented by LAD) [38] to 92.0% (STG autograft ACLR augmented by woven polyester) [48].
- ACL‐RSI survey score
  Three non‐comparative studies [13, 21, 22] reported the ACL‐RSI survey score. Two studies [21, 22] evaluated InternalBrace augmented or LARS augmented hamstring autograft ACLR; the mean ACL‐RSI scores at 24‐month follow‐up were 78.0 (95% CI: 76.9–79.1) and 74.8 (95% CI: 68.5–81.1) respectively. Another study [13] reported the six‐item short version ACL‐RSI survey score; the mean was 79.8/100 (95% CI: 75.8–83.8) at follow‐up (mean: 37.9 months) after quadriceps tendon, bone‐patellar tendon‐bone, quadrupled semitendinosus autograft or allograft ACLR with InternalBrace augmentation.
- PROs
  PROs for five outcomes are reported below:
  1. KOOS score
  Six comparative studies [3, 8, 17, 42, 53, 74] with mid‐term follow‐up reported KOOS scores. Five studies [3, 8, 17, 42, 74] reporting KOOS scores for each domain were included in a meta‐analysis, and one comparative study [53] was excluded from the meta‐analysis due to insufficient data. Meta‐analysis results, with low‐certainty evidence, showed statistically significant, but no clinically meaningful benefit of SA compared to traditional ACLR (MD: 2.79, 95% CI: 0.99–4.59, *N* = 627; *I* ^2^ = 79.1%; *p* < 0.01; Supporting Information S1: Figure S7). Two studies [14, 16] compared KOOS scores at the long‐term follow‐up and showed no benefit of SA (MD: 0.83, 95% CI: −0.71 to 2.37, *N* = 218; *I* ^2^ = 0%; *p* = 0.29; Supporting Information S1: Figure S8).
  Additionally, one non‐comparative study on LARS‐augmented ACLR [21] reported a mean KOOS QoL score at 2‐year follow‐up of 82.4 (95% CI: 77.63–87.17), and mean scores of over 90 for all other four KOOS domains. Three non‐comparative studies on InternalBrace‐augmented ACLR [15, 26, 62] reported a mean overall KOOS score of over 85.
  2. Tegner activity score
  Nine comparative studies [3, 4, 30, 36, 44, 52, 56, 59, 72] reported Tegner activity scores at the mid‐term follow‐up. Pre‐operative Tegner activity scores were not reported in all studies, limiting our meta‐analysis from calculating mean differences in improvement scores between the groups. Our analysis, with moderate‐certainty evidence, showed no substantial benefit of SA over the control in the post‐operative Tegner activity score, although it was statistically significant (MD: 0.30; 95% CI: 0.05–0.56; *N* = 816; *I* ^2^ = 28.65%; *p* = 0.02; Supporting Information S1: Figure S9). Three studies reported Tegner activity scores at long‐term follow‐up but showed no benefit of SA (MD: 0.25; 95% CI: −0.49 to 1.00; *N* = 258; *I* ^2^ = 57.27%; *p* = 0.51; Supporting Information S1: Figure S10).
  Furthermore, six non‐comparative studies [12, 15, 21, 22, 24, 63] reported Tegner activity score at the mid‐term follow‐up. The mean scores were 7.0 (SD: 2.30 [63] to 2.37 [15]) to 7.10 (SD: 1.50) [22] for patients undergoing ACLR augmented by FiberTape (Arthrex) using the InternalBrace technique and 5.78 (SD: 1.20) [12] to 7.5 (SD: 1.60) [21] for patients undergoing ACLR augmented by other synthetic materials. One non‐comparative study reported Tegner activity score at the long‐term follow‐up to be 6.00 (SD: 2.00) for patients undergoing InternalBrace augmented ACLR [75].
  3. Lysholm score
  Eleven comparative studies [3, 4, 36, 41, 44, 52, 56, 59, 71, 72, 76] reported outcomes for the Lysholm scores in the mid‐term follow‐up. Similarly to the Tegner activity score, pre‐operative Lysholm scores were not provided. Moderate‐certainty evidence showed patients undergoing ACLR with SA experience no benefit of post‐operative Lysholm score compared to the control group (MD: 0.19, 95% CI: −2.46 to 2.84; *N* = 1001; *I* ^2^ = 88.61%; *p* = 0.89; Supporting Information S1: Figure S11). One comparative study [60] reported Lysholm score at short‐term follow‐up and found no significant difference (MD = 0.06, *p* = 0.76) in post‐operative Lysholm score between ACLR with or without fibre tape augmentation. Also, nine non‐comparative studies [12, 13, 15, 20, 21, 22, 24, 63, 68] reported mid‐term follow‐up of Lysholm scores. The mean score for the ACLR using an SA ranged from 87.10 (ACLR augmented by LARS) [24] to 96.50 (ACLR augmented by FiberTape (Arthrex) using the InternalBrace technique) [22].
  4. IKDC score
  Seven studies [3, 8, 41, 52, 71, 72, 76] presented IKDC score. A non‐significant difference of 3.06 in the IKDC score was found, with high statistical heterogeneity and low‐certainty evidence (95% CI: −1.52 to 7.63; *N* = 741; *I* ^2^ = 93.23%; *p* = 0.19; Supporting Information S1: Figure S12). Additionally, five non‐comparative studies [15, 21, 24, 63, 68] reported mid‐term IKDC scores. The mean post‐operative IKDC score was 83.40 [68] when polyethylene terephthalate (Trevira) was used as SA. The mean post‐operative IKDC score ranged from 88.6 (SD: 12.05) [15] to 93.1 (SD: 9.06) [63] when InternalBrace (Arthrex) was used, and ranged from 86.5 (SD: 11.6) [24] to 91.6 (SD: 8.3) [21] when LAD was used for ACLR SA.

**Table 1:** Summary of findings.

Graft synthetic augmentation (SA) for patients undergoing ACLR					
Patient or population: Patients undergoing ACLR					
Intervention: ACLR with SA					
Comparison: ACLR without SA					
Outcomes		Effect size; OR/MD (95% CI)	No. of patients (studies)	Certainty of the evidence (GRADE)	Comments
Graft failure	Mid‐term	OR: 0.70 (0.41–1.17)	1746; 802 with and 944 without SA [16]	⊕⊕⊕ 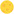 moderate	OR less than 1 suggests lower graft failure with augmentation, driven by Internal Brave.
	Long‐term	OR: 0.55 (0.19–1.60)	479; 238 with and 241 without SA [6]	⊕⊕⊕ 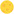 moderate	OR more than 1 suggests higher graft failure with augmentation, Kennedy LAD.
Return‐to‐sport (RTS)	Mid‐term	OR: 1.58 (1.12–2.22)	716; 294 with and 422 without SA [8]	⊕⊕⊕ 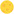 moderate	OR greater than 1 suggests a higher RTS with augmentation.
	Long‐term	OR: 1.68 (0.70–4.08)	515; 256 with and 259 without SA [5]	⊕⊕⊕ 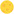 moderate	
Patient‐reported outcomes (PROs)					
1. Tegner	Mid‐term	MD: 0.30 (0.05–0.56)	816; 341 with and 475 without SA [9]	⊕⊕⊕ 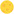 moderate	A higher score means a better activity level.
	Long‐term	MD: 0.25 (−0.49 to 1.00)	258; 111 with and 147 without SA [3]	⊕⊕ 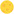 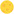 low	A higher score means a better activity level.
2. Lysholm; Mid‐term		MD: 0.19 (−2.46 to 2.84)	1001; 442with and 559 without SA [11]	⊕⊕⊕ 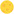 moderate	A higher score means better knee function.
3. IKDC; Mid‐term		MD: 4.94 (−0.37 to 10.24)	741; 337 with and 416 without SA [7]	⊕⊕ 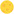 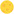 low	A higher score means better knee function.
4. KOOS	Mid‐term	MD: 2.79 (0.99–4.59)	627; 280 with and 347 without SA [5]	⊕⊕ 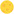 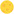 low	A higher score means better knee condition and function.
	Long‐term	MD: 0.83 (−0.71 to 2.37)	218; 88 with and 130 without SA [2]	⊕⊕⊕ 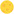 moderate	A higher score means better knee condition and function.

*Note*: GRADE Working Group grades of evidence.

High certainty: Further research is very unlikely to change our confidence in the estimate of effect.

Moderate certainty: Further research is likely to have an important impact on our confidence in the estimate of effect and may change the estimate.

Low certainty: Further research is very likely to have an important impact on our confidence in the estimate of effect and is likely to change the estimate.

Very low certainty: The authors of this review are very uncertain about the estimate.

Abbreviations: ACLR, anterior cruciate ligament reconstruction; CI, confidence interval; IKDC, International Knee Documentation Committee; KOOS, Knee injury and Osteoarthritis Outcome Score; MD, mean difference; OR, odds ratio.

### Sensitivity analysis

Alternatively, dividing subgroups based on the year of publication showed no significant difference in graft failure rate between SA ACLR and traditional ACLR in the mid‐term, and yielded similar results in the long‐term, with InternalBrace being significantly beneficial. Recent studies (within 10 years) showed a significantly higher RTS rate in the SA group than the control group in the mid‐term (OR: 1.86; 95% CI: 1.12–2.22; *p* < 0.01; Supporting Information S1: Figure A3), while long‐term results remained similar to the base‐case. Differences in PROs remained clinically and/or statistically non‐significant, which is similar to the base‐case results. Details of the sensitivity analysis are available in Supporting Information S2: Appendix 4, Figures A1–A7.

## DISCUSSION

The most important finding of the present study was that a meta‐analysis of comparative studies with moderate‐certainty evidence showed a high rate of RTS for the SA group in the mid‐term follow‐up. A reduced graft failure rate in the long term was observed only with the InternalBrace technique. Patients undergoing ACLR with SA experienced greater KOOS scores, but the differences were not clinically meaningful. Additionally, our findings indicated higher post‐operative scores in the Tegner activity scale in the SA group, but this was also not clinically meaningful. There was also no benefit in Lysholm and IKDC scores for the SA ACLR group. These findings are based on available data, though not all studies reported every outcome. The quality assessment highlighted a moderate to serious risk of bias in the non‐randomised studies, primarily due to confounding factors and incomplete data reporting, pointing to a lack of high‐quality research in this field.

Our meta‐analysis found a non‐significant reduction in graft failure for ACLR with SA compared to ACLR alone across all SA techniques. Subgroup analysis showed a significant decrease in graft failure with InternalBrace compared to traditional ACLR in the long‐term, but no significant benefit with other SA. Our results are in line with the reviews by Zheng et al. [77] and Gao et al. [25] that showed the overall failure rate of suture tape‐augmented grafts ranged from 0.0% to 6.7% [77], and 0.0% to 8.1% [25] respectively. However, Dhillon et al. [18] found a higher graft failure rate in the SA group ranging from 1.0% to 25.0% [18]. This difference could be due to different interpretations of graft failure by review authors. Graft re‐rupture (and/or revision ACLR) were interpreted as graft failure in this review, while Dhillon et al. [18] used data from total reoperations in some of their included studies [52, 74]. The results of synthesising non‐comparative studies showed a low rate of graft failure below 8.7% [26] for patients undergoing InternalBrace‐augmented ACLR, which aligned with the results from the comparative studies.

Our meta‐analysis with moderate‐certainty evidence indicated a significant improvement in the RTS rate for SA ACLR, especially using the InternalBrace technique, suggesting these patients were more likely to resume sports activities. This is in line with the finding of the review by Gao et al. [25] which found a significantly higher RTS rate for suture (tape) augmented ACLR. The review by Zheng et al. [77] also reported that adding suture tape to ACLR was associated with a trend towards significantly better RTS, while Dhillon et al. [18] reported that no studies using the InternalBrace found a significant between‐group difference for RTS, potentially due to inclusion of an additional study [52].

Results of the PROs meta‐analysis, with low‐ to moderate‐certainty evidence, showed a statistically significant difference in the post‐operative Tegner activity levels and KOOS scores for the SA group, but these did not meet the minimal clinically important difference thresholds [7]. This effect was driven by the Infinity‐Lock Neoligament and InternalBrace, while Kennedy LAD and LARS showed no significant benefit. Results, also with low‐certainty evidence, showed no benefit in Lysholm and IKDC scores for SA ACLR. This suggests that while SA may increase activity levels and knee stability statistically, it is unlikely to result in a substantial clinical improvement after ACLR, based on commonly used PROs. Overall, our results of PROs were consistent with previous reviews [18, 25, 77]. One non‐comparative study [22] found that the post‐operative Tegner level had become higher than the pre‐injury level, suggesting good outcomes of adding InternalBrace augmentation to ACLR.

### Strengths

This review employed a comprehensive search strategy across a broad range of electronic databases with no date restrictions to identify all relevant studies. While older studies may involve outdated techniques and materials, the subgroup analyses in this review, particularly by augmentation material, help distinguish findings from different SA technologies, maintaining relevance to modern clinical practice.

Existing evidence was reviewed by including both comparative and non‐comparative studies on different types of synthetic material. A valid methodological approach was applied, using Hedges' *g* for effect size estimation and *I* ^2^ for heterogeneity assessment, ensuring statistical rigour in comparative analyses while maintaining transparency in reporting narrative findings from non‐comparative studies. This review included a larger number of patients (4289; 2729 from comparative studies and 1560 from non‐comparative studies) compared to previous reviews (314 [77], 528 [18] and 851 [25]), allowing for a broader assessment of SA's effectiveness across different populations.

### Limitations

Results should be interpreted cautiously. Thirty‐five [2, 3, 4, 8, 13, 14, 15, 16, 17, 20, 21, 22, 23, 24, 26, 36, 38, 40, 42, 48, 50, 52, 55, 56, 57, 59, 63, 65, 68, 69, 71, 72, 74, 75, 76] out of 47 studies were Level of Evidence 3 or 4, introducing biases due to lack of randomisation or comparison groups. Whilst meta‐analysis was appropriate with only some but limited study heterogeneity, therefore justifying the application of a random‐effects meta‐analysis model to account for statistical heterogeneity, some variations in study design and outcome reporting remained. Therefore, our findings should be caveated with this matter. Additionally, the exclusion of non‐English publications and cadaveric studies may have limited the diversity and generalisability of our findings. Similarly, while searching published and unpublished evidence sources, these were predominantly Western, English language databases, thereby potentially limiting the inclusion of a global evidence perspective. Finally, 18 studies [8, 14, 15, 16, 17, 21, 22, 23, 24, 26, 36, 44, 50, 53, 62, 63, 74, 75] reported conflicts of interest or funding from SA manufacturers.

### Clinical relevance

This comprehensive review showed that SA has been used for a long time in various forms, and is safe overall, with some potential benefits. Contemporary techniques, in particular, showed an improvement in RTS rates, and InternalBrace may result in reduced re‐rupture rates. Both of these outcomes have a direct benefit for patients. SA could be considered for all patients but may be particularly beneficial for patients at high risk of re‐rupture and those wanting to return to a high level of sports.

### Research priorities and uncertainties

To address the evidence gaps identified, future studies should focus on conducting rigorous randomised controlled trials, standardising outcome measures, and exploring the influence of patient‐specific factors. These efforts will help clarify the role of SA in ACLR and enhance clinical decision‐making.

## CONCLUSION

Evidence from this review suggests that patients undergoing ACLR with SA may experience better RTS outcomes compared to those undergoing ACLR alone. Additionally, enhancing ACLR with InternalBrace augmentation may help reduce graft failure rates in the long term. However, the evidence of SA's impact on PROs remains inconclusive. Recommendations are conservative due to the evidence base's moderate to serious risk of bias. Addressing these uncertainties through further high‐quality research will be crucial in determining the full clinical benefits and potential risks associated with SA in ACLR.

## AUTHOR CONTRIBUTIONS

Andrew Metcalfe, Hema Mistry, Nick Smith, Seyran Naghdi and Toby Smith developed the study design. Seyran Naghdi conducted the literature searches. Nick Smith, Seyran Naghdi, Toby Smith and Yufei Jiang screened the literature. Yufei Jiang extracted data from the articles. Seyran Naghdi ensured the accuracy and completeness of the data extraction. Seyran Naghdi and Yufei Jiang performed the quality assessment independently. Seyran Naghdi performed a meta‐analysis of the data, which was cross‐checked by Yufei Jiang. Yufei Jiang conducted a narrative analysis of the data, which was cross‐checked by Seyran Naghdi. Yufei Jiang and Seyran Naghdi wrote the first draft of the manuscript, which all authors revised. All authors reviewed and agreed with the final version. All authors had access to all the data in the study and had final responsibility for the decision to submit for publication.

## CONFLICT OF INTEREST STATEMENT

Nick Smith has been paid by Arthrex for presenting on meniscal root repair techniques at an educational event and received travel expenses to an Arthrex conference. Andrew Metcalfe is both lead and co‐investigator on multiple grants from the UK National Institute for Health Research (NIHR), but these are not related to this review. He is an investigator on three NIHR‐funded trials (START:REACTS, RACER‐Knee and RACER‐Hip, Andrew Metcalfe leads START:REACTS and RACER‐Knee), for which Stryker have funded treatment costs and some imaging costs. These are not related to the review and for all of these studies, the full independence of the study team is protected by legal agreements. The remaining authors declare no conflicts of interest.

## ETHICS STATEMENT

The ethics statement is not available.

## Supporting information

Supporting information.

Supporting information.

## Data Availability

The data sets used and/or used during the current study are available from the corresponding author upon reasonable request.
