# Supplementary material for: Synthetic augmentation in ACL reconstruction may reduce re‐rupture rates and increase return‐to‐sport rates: A systematic review and meta‐analysis
Source: Knee Surg Sports Traumatol Arthrosc. 2025 Apr 18;34(2):424–35. doi: 10.1002/ksa.12680 (PMC12850593; doi:10.1002/ksa.12680)
Supplement: Supplementary file 1 — Supporting information. [file KSA-34-424-s001.docx]

**Figure S1: ROB2 risk of bias assessment**


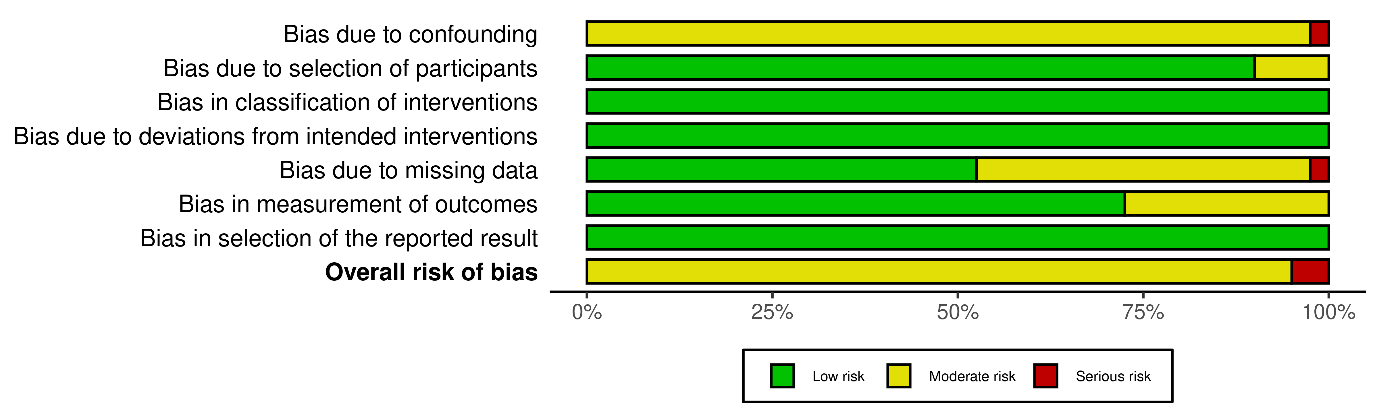


**Figure S2: ROBINS-I risk of bias assessment**

**Table S1: Studies characteristics**

| **Study** | **Level of evidence** | **Intervention/ comparator** | **Number of participants** | **Age (mean ±SD)** | **Age (range)** | **Mean (if not other specified) follow-up, month** | **% Female** | **BMI** |
| --- | --- | --- | --- | --- | --- | --- | --- | --- |
| **Comparative studies** | | | | | | | | |
| Roth et al., 1985(32) | 3 | ACLR + LAD | 38 | 29 | 18-46 | 50 | 37.78% | NI |
|  |  | ACLR | 45 | 32 | 21-46 | 64 | 28.95% | NI |
| Moyen et al., 1992(24) | 1 | ACLR + LAD | 34 | 24 | NI | 34 | 41.18% | 22.8 |
|  |  | ACLR | 30 | 24 | NI |  | 40.00% | 22.4 |
| Noyes et al., 1992(29) | 3 | ACLR + LAD | 46 | 24 | 14-43 | 30 | 41.30% | NI |
|  |  | ACLR | 64 | 24 | 14-40 | 37 | 35.94% | NI |
| Sgaglione et al., 1992(35) | 3 | ACLR + LAD | 15 | 26.5 ± 8.2 | 16-46 | 31.2 | 40.00% | NI |
|  |  | ACLR | 28 | 24.8 ± 6.6 | 15-38 | 34.1 | 28.57% | NI |
| Barrett et al., 1993(3) | 3 | ACLR + LAD | 25 | 25 | 14-42 | 24 | 32.00% | NI |
|  |  | ACLR | 50 | 23 | 14-39 | 24 | 18.00% | NI |
| Santi et al., 1994(33) | 3 | ACLR + LAD | 28 | 27.3 ± 7.0 | NI | 31.4 | 25.00% | NI |
|  |  | ACLR | 32 | 24.3 ± 7.3 | NI | 32.3 | 28.13% | NI |
| Muren et al., 1995(27) | 1 | ACLR + LAD | 20 | 25 | 18-36 | 48 | 35.00% | NI |
|  |  | ACLR | 20 | 23 | 18-32 | 48 | 20.00% | NI |
| Grøntvedt et al., 1996(17) | 1 | ACLR + LAD | 49 | 26 | 16-48 | 24 | 55.00% | NI |
|  |  | ACLR | 51 |  |  |  |  |  |
| Marcacci et al., 1996(21) | 3 | ACLR + LAD | 60 | 24 | 15-43 | 69 | 20.00% | NI |
|  |  | ACLR | 25 | 23 | 15-38 | 69 | 16.00% | NI |
| Thuresson et al., 1996(44) | 1 | ACLR + LAD | 45 | 26.7 | 16-46 | Minimum 24.  Range 24-49. | 28.05% | NI |
|  |  | ACLR | 37 |  |  |  |  |  |
| Steenbrugge et al., 2002^a (39)^ | 3 | ACLR + LAD | 25 | 37 | 23-55 | 74 | 20.00% | NI |
|  |  | ACLR | 29 | 39 | 25-63 | 94 | 41.18% | NI |
| Muren et al., 2003(26) | 1 | ACLR + LAD | 20 | 25 | 19-44 | 84 | 35.00% | NI |
|  |  | ACLR | 20 | 25 | 20-33 | 84 | 50.00% | NI |
| Aujla et al., 2021(2) | 3 | ACLR + LARS | 66 | 26.8 ± 9.5 | NI | 24 | 33.33% | 24.7 ± 3.7 |
|  |  | ACLR | 130 | 27.5 ± 8.6 | NI | 24 | 39.23% | 25.3 ± 3.2 |
| Ebert et al., 2022(14) | 3 | ACLR + LARS | 67 | 31.1 ± 9.3 | 16-49 | Minimum 84 | 32.84% | 26.2 ± 3.1 |
|  |  | ACLR | 69 | 30.8 ± 10.6 | 16–49 | Minimum 84 | 36.23% | 26.0 ± 3.1 |
| Zhang et al., 2022(47) | 3 | ACLR + LARS | 36 | 27 | 17-37 | 12 | 16.67% | NI |
|  |  | ACLR | 32 | 33 | 21-42 | 12 | 26.67% | NI |
| Bodendorfer et al., 2019(4) | 3 | ACLR + IB | 30 | 29.34 ± 7.55 | 18- 49 | 29.0 | 56.6% | 25.79 |
|  |  | ACLR | 30 | 29.65 ± 5.65 | 18- 42 | 30.08 | 56.6% | 26.74 |
| Shantanu et al., 2019(36) | 2 | ACLR + IB | 25 | 27.84 ± 9.44 | 16-53 | 6 | 16% | NI |
|  |  | ACLR | 25 | 32.16 ± 9.22 | 17-56 | 6 | 20% | NI |
| Parkes et al., 2021(30) | 3 | ACLR + IB | 36 | 25.3 ± 8.6 | 13- 44 | 26.1 | 31% | 25.9 |
|  |  | ACLR | 72 | 24.9 ± 9.6 | 13- 54 | 31.3 | 31% | 25 |
| von Essen et al., 2022(45) | 3 | ACLR + IB | 40 | 29.15 | 17- 48 | 24.0 | 42.5% | NI |
|  |  | ACLR | 40 | 29.15 | 17- 48 | 24.0 | 42.5% | NI |
| Kitchen et al., 2022(19) | 3 | ACLR+IB | 40 | 15.7 | 9.5- 19 | 27.6 | 52.5% | NI |
|  |  | ACLR | 40 | 14.9 | 9- 19 | 29.0 | 55% | NI |
| Daniel et al., 2023^b^ (10) | 3 | ACLR + IB | 100 | 19.0 ± 5.61 | NI | 33.4 | 51% | 25.5 |
|  |  | ACLR | 100 | 19.9 ± 5.61 | NI | 48.6 | 51% | 26.1 |
| Daniel and Smith, 2024 (1)(7) | 3 | ACLR + IB | 52 | 18.6 ± 3.31 | NI | 60 | 38% | 26.6 |
|  |  | ACLR | 62 | 17.7 ± 3.21 | NI | 62.4 | 56% | 27.0 |
| Daniel and Smith, 2024 (2)(9) | 3 | ACLR + IB | 36 | 20.1 ± 5.82 | 13-39 | 50.4 | 31% | 24.4 |
|  |  | ACLR | 68 | 21.5 ± 7.15 |  | 61.2 | 53% | 25.6 |
| Meng et al., 2024(22) | 1 | ACLR + IB | 20 | 33.7 ± 8.8 | NI | 24 | 25% | 25.4 |
|  |  | ACLR | 20 | 36.4 ± 11.0 | NI | 24 | 25% | 25.2 |
| Darestani et al., 2023(42) | 3 | ACLR + FiberWire | 90 | 30.5 ± 7.6 | NI | 24 | 2.2% | 26.3 |
|  |  | ACLR | 79 | 31.6 ± 8.3 | NI | 24 | 8.9% | 27.5 |
| Tensho et al., 2024(43) | 3 | ACLR + FiberWire | 53 | 29.3 ± 14.7 | 12-65 | 25.9 | 58.49% | NI |
|  |  | ACLR | 53 | 28.1 ± 13.6 | 14-70 | 25.5 | 56.60% | NI |
| Mohan et al., 2023(23) | 3 | ACLR + Neoligament | 70 | 34.5 | 19-50 | 20 | 26% | 27.7 |
|  |  | ACLR | 111 | 30.4 | 18- 55 | 20 | 21% | 28.5 |
| Peterson et al., 2014(31) | 1 | ACLR + poly(urethane urea) | 96 | 27 ± 8 | 16-47 | 12, 48, and 140 | 37% | NI |
|  |  | ACLR | 105 | 27 ± 7 | 15- 47 | 12, 48, and 140 | 47% | NI |
| **Non-comparative studies** | | | | | | | | |
| Dahlstedt et al., 1990(5) | N/A | ACLR + LAD | 23 | 26.74 ± 5.58 | 19-42 | 36.57 | 30.43% | NI |
| MacDonald et al., 1995(20) | 4 | ACLR + LAD | 40 | 23.5 | NI | 20-33 | 15.00% | NI |
| Saragaglia et al., 1995(34) | 4 | ACLR + LAD | 107 | 31.9 | 19-55 | 66 | 32.71% | NI |
| Asahina et al., 1996(1) | 4 | ACLR + LAD | 44 | 23 | NI | 38 | 63.64% | NI |
| Kdolsky et al., 1997(18) | N/A | ACLR + LAD | 315 | NI | NI | 46.8 or 88.8 | NI | NI |
| Muneta et al., 2000(25) | N/A | ACLR + LAD | 60 | NI | NI | 94.7 | 66.67% | NI |
| Falconer et al., 2015(15) | 4 | ACLR + LAD | 111 | 33.8 | 16-54 | 29 | 34.23% | NI |
| Ebert et al., 2019(12) | 4 | ACLR + LAD | 50 | 26.3 ± 9.6 | 16-49 | 24 | 36.00% | 24.8 ± 4.0 |
| Duong et al., 2022(11) | 4 | ACLR + IB | 37 | 33 ± 7.84 | 19- 48 | 12 | 24.3% | NI |
| Ebert et al., 2023(13) | 4 | ACLR + IB | 53 | 28.1 ± 9.2 | 16- 45 | 24 | 43.4% | 24.9 |
| Wilson et al., 2023(46) | 4 | ACLR + IB | 97 | 34.7 ± 13.4 | NI | 60 | 23.71% | NI |
| Daniel et al., 2024(6) | 4 | ACLR + IB | 252 | 23.6 ± 12.15 | NI | 37.9 | 49.6% | 26.1 |
| Smith et al., 2024(38) | 4 | ACLR + IB | 25 | 19.9 ± 6.63 | NI | 28 | 64% | 25 |
| Simard et al., 2024(37) | N/A | ACLR + IB | 66 | 37.2 | NI | 24 | 38% | NI |
| Daniel and Smith, 2025(8) | 4 | ACLR + IB | 60 | 16.8 | 13-23 | 37.1 | 68% | 24.1 |
| Garside et al., 2025(16) | 4 | ACLR + IB | 23 | 20.3 ± 3.5 | 15-25 | 30 | 52.2% | NI |
| Takazawa et al., 1996(41) | 3 | ACLR + Telos ligament | 121 | 21.43 | NI | 56.5 | 0% | 25.71 |
| Nakayama et al., 2000(28) | 4 | ACLR + Woven polyester | 50 | 24.3 | 19- 39 | 12 | 50% | NI |
| Struewer et al., 2013(40) | 4 | ACLR + Polyethylene terephthalate | 126 | 32 | 19- 60 | 29 | 39.68% | NI |

**Note:** SD, standard deviation; NI, no information; ACLR, anterior cruciate ligament reconstruction; IB, InternalBrace; LARS: Ligament Augmentation and Reconstruction System; LAD: Ligament Augmentation Device; BMI, body mass index.

Comparative studies that compared SA ACLR with other interventions are categorised as non-comparative studies and only data in the SA ACLR groups was used. Level of evidence not applicable (N/A).

a: In this study, patient characteristics data was only available for the 40 patients who reported PROs. b: In this study, we picked 2y follow-up data to be more comparable to other studies.


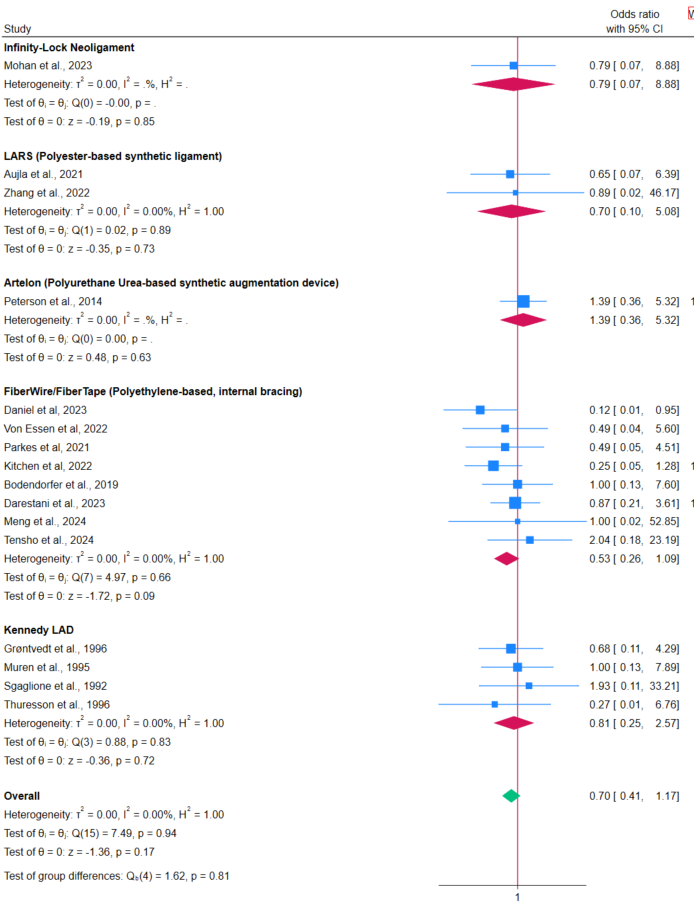


**Figure S3: Forest plot for graft failure rate; Mid-term follow-up; Subgroup of different materials**. **Effect size reported in odds ratio and 95% CI**


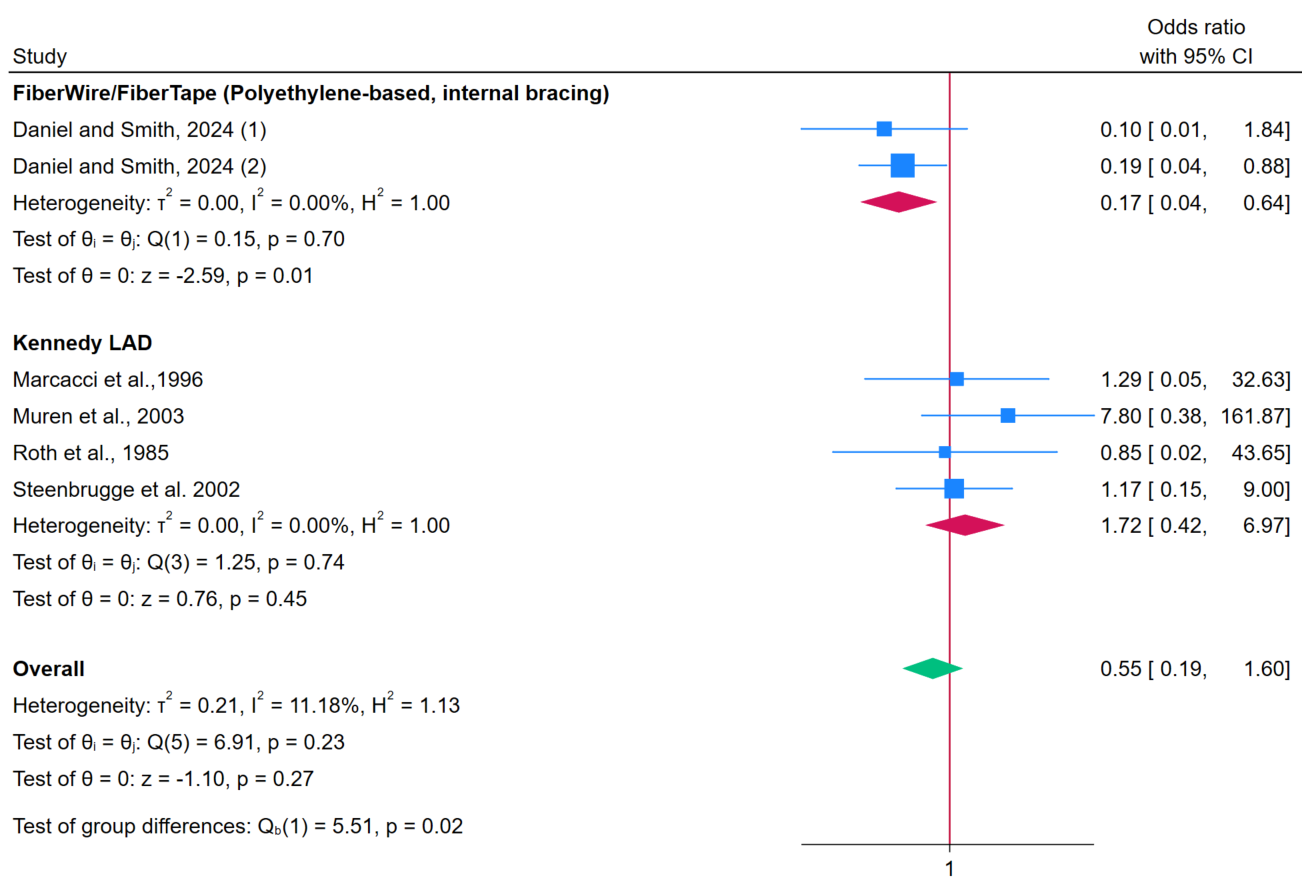


**Figure S4: Forest plot for graft failure rate; Long-term follow-up; Subgroup of different materials; Effect size reported in odds ratio and 95% CI**


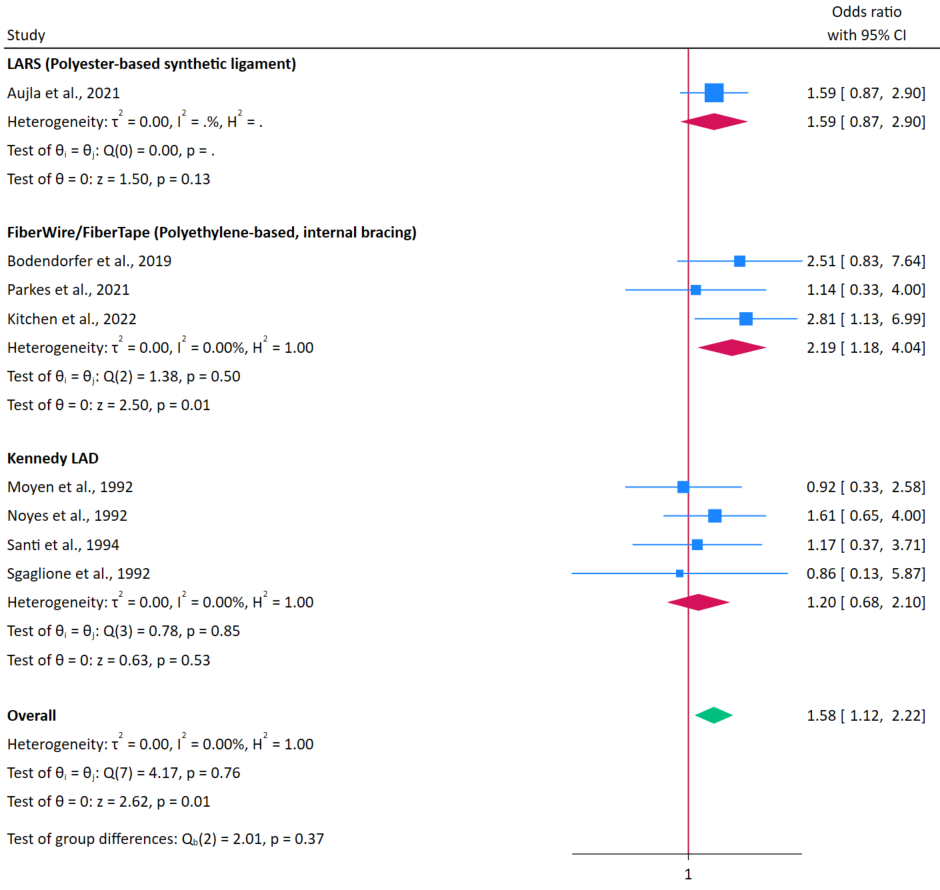


**Figure S5: Forest plot for return to sport rate; Mid-term follow-up; Subgroup of different materials;** **Effect size reported in odds ratio and 95% CI**


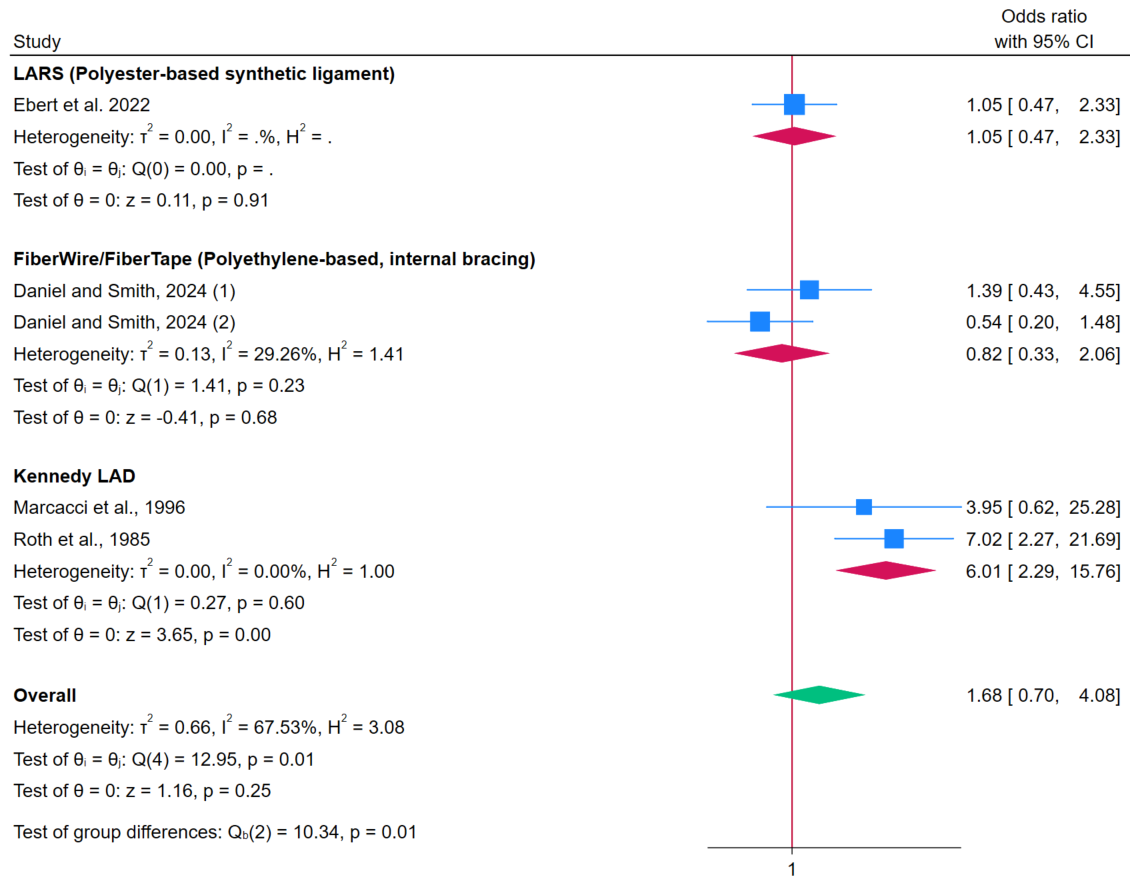


**Figure S6: Forest plot for return to sport rate; Long-term follow-up; Subgroup of different materials**. **Effect size reported in odds ratio and 95% CI**


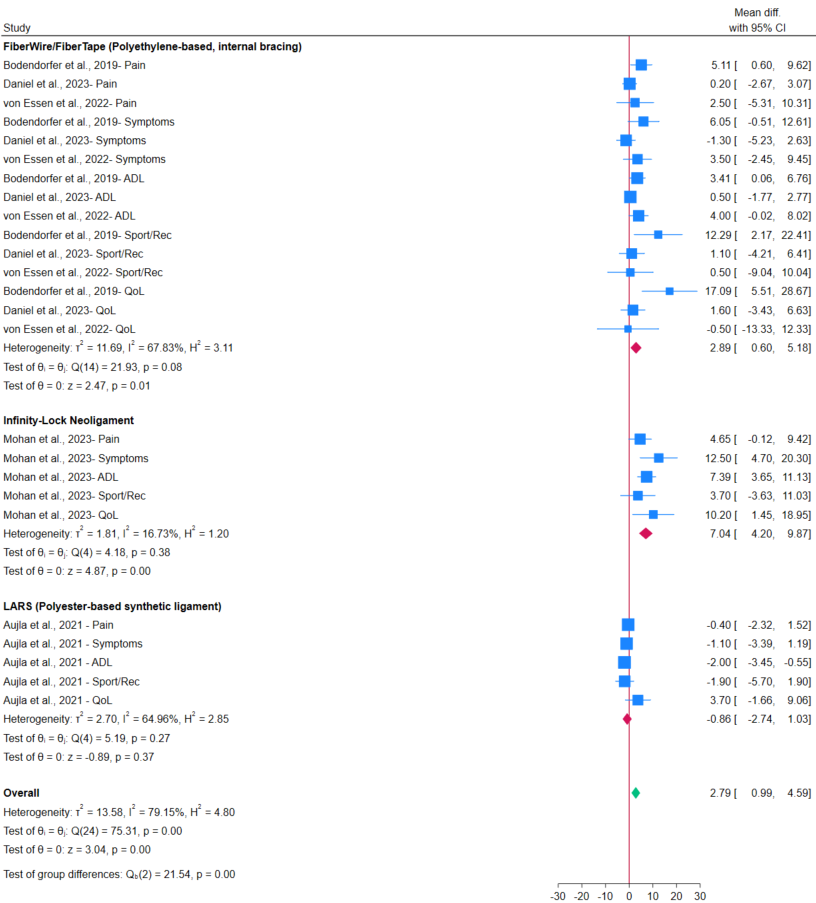


**Figure S7: Forest plot for post-operative KOOS score- Mid-term follow-up**; **Subgroup of different materials.** **Effect size reported in mean difference and 95% CI**


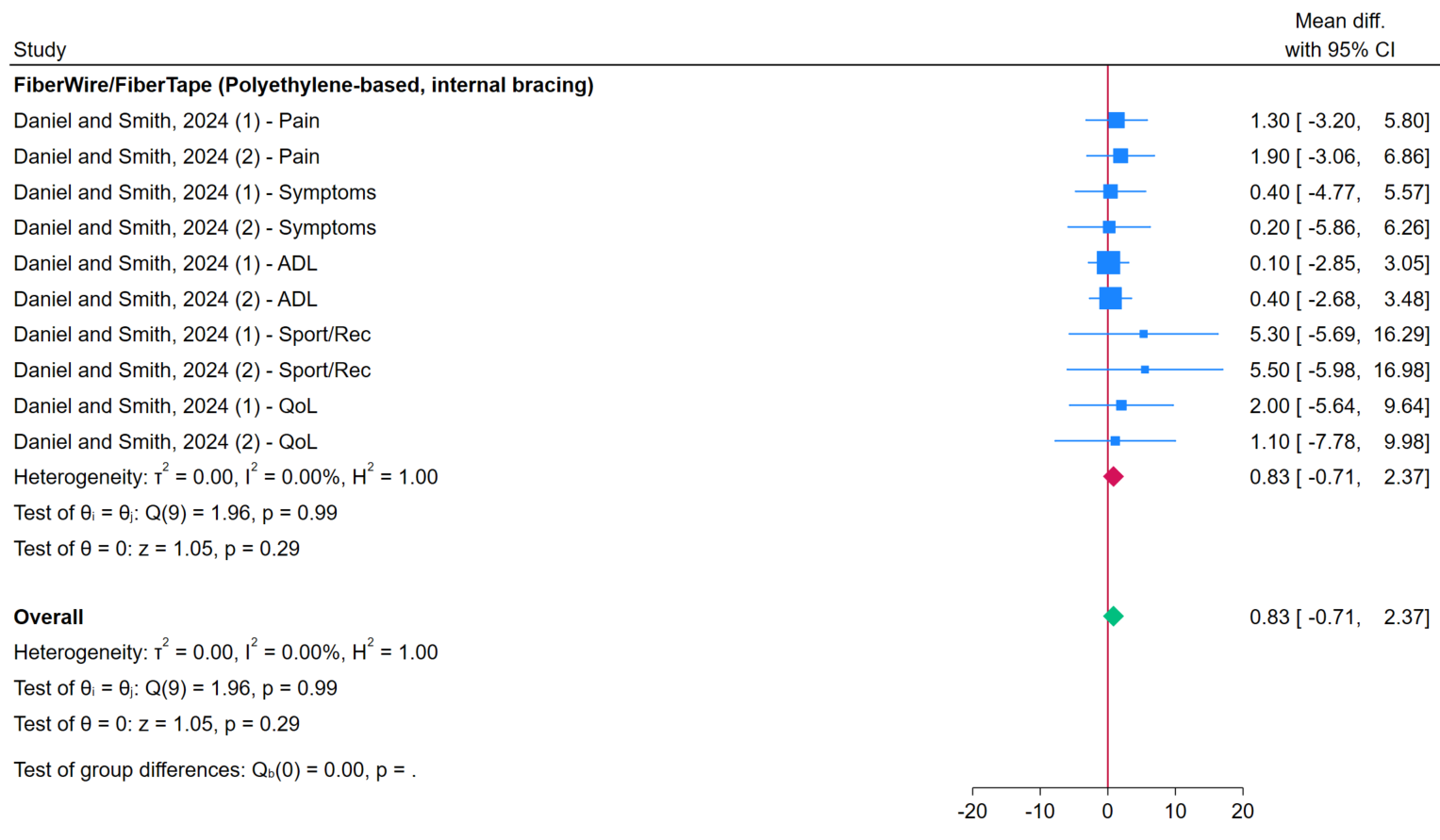


**Figure S8: Forest plot for post-operative KOOS score- Long-term follow-up**; **Subgroup of different materials.** **Effect size reported in mean difference and 95% CI**


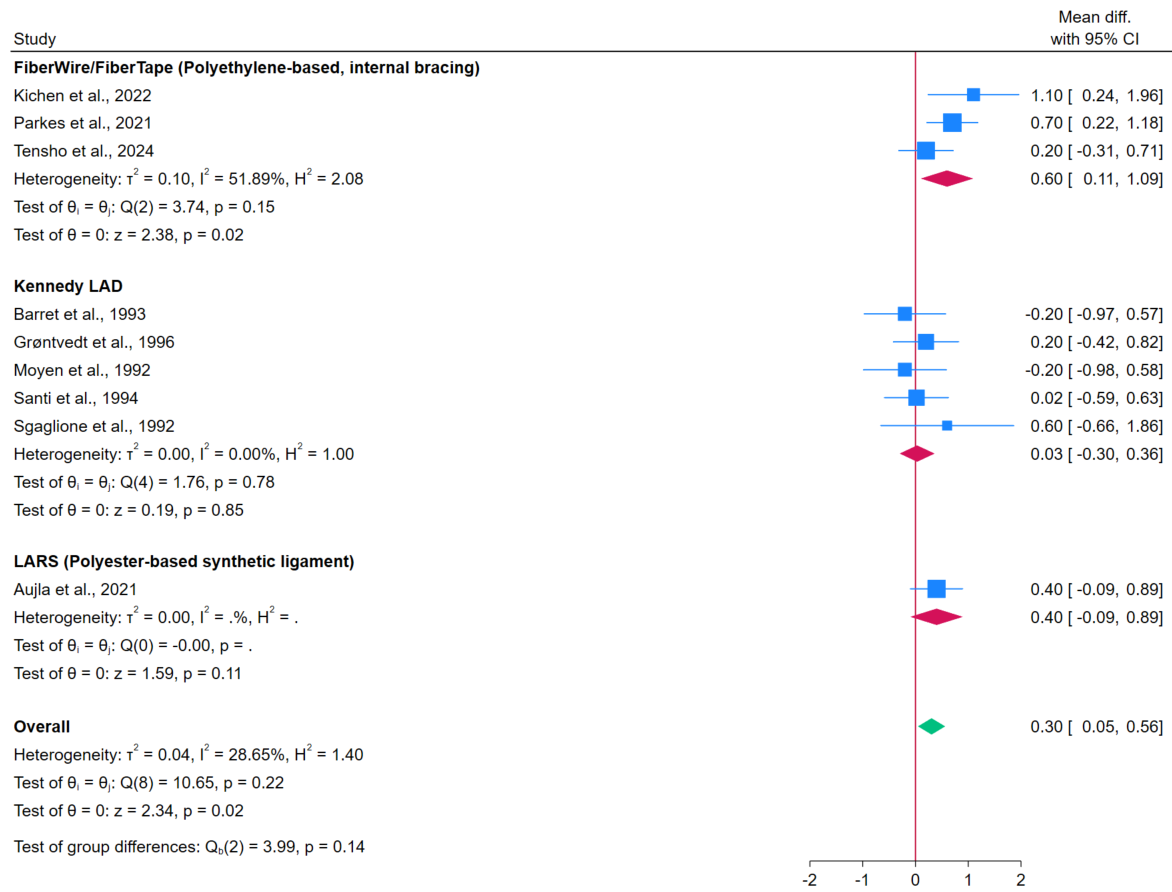
**Figure S9: Forest plot for postoperative Tegner activity score; Mid-term follow-up; Subgroup of different materials.** **Effect size reported in mean difference and 95% CI**


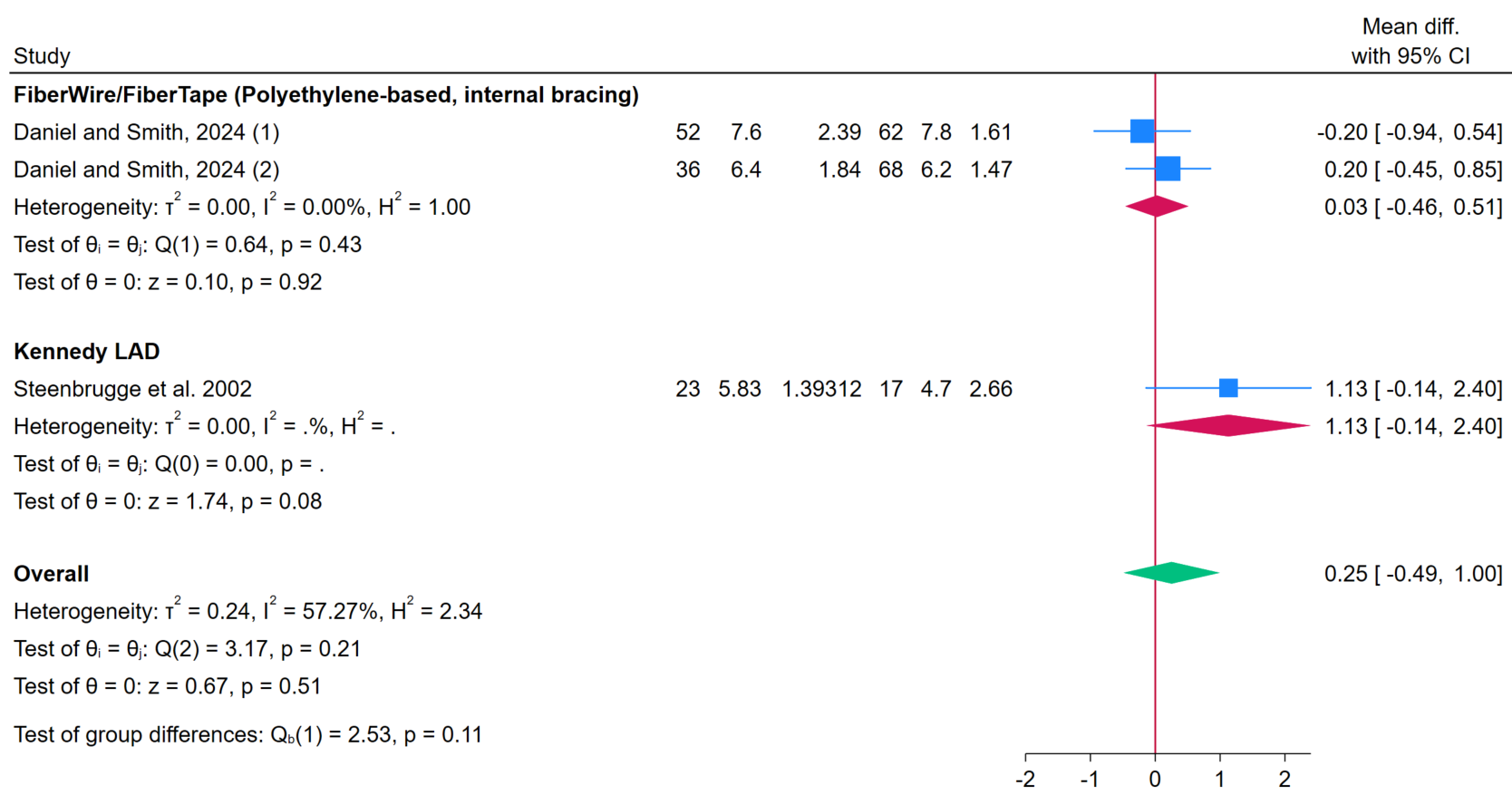


**Figure S10: Forest plot for postoperative Tegner activity score; long-term follow-up; Subgroup of different materials.** **Effect size reported in mean difference and 95% CI**


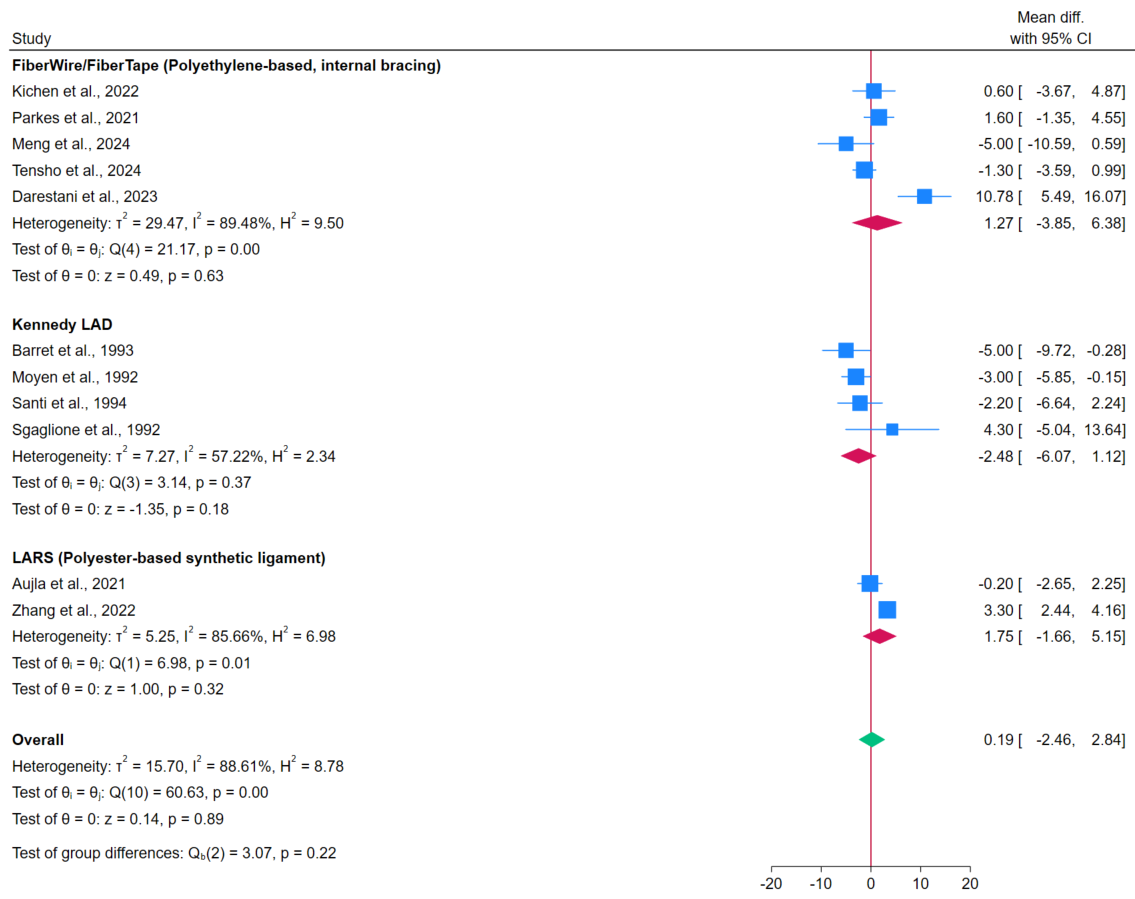


**Figure S11: Forest plot for postoperative Lysholm; Mid-term follow-up; Subgroup of different materials**. **Effect size reported in mean difference and 95% CI**


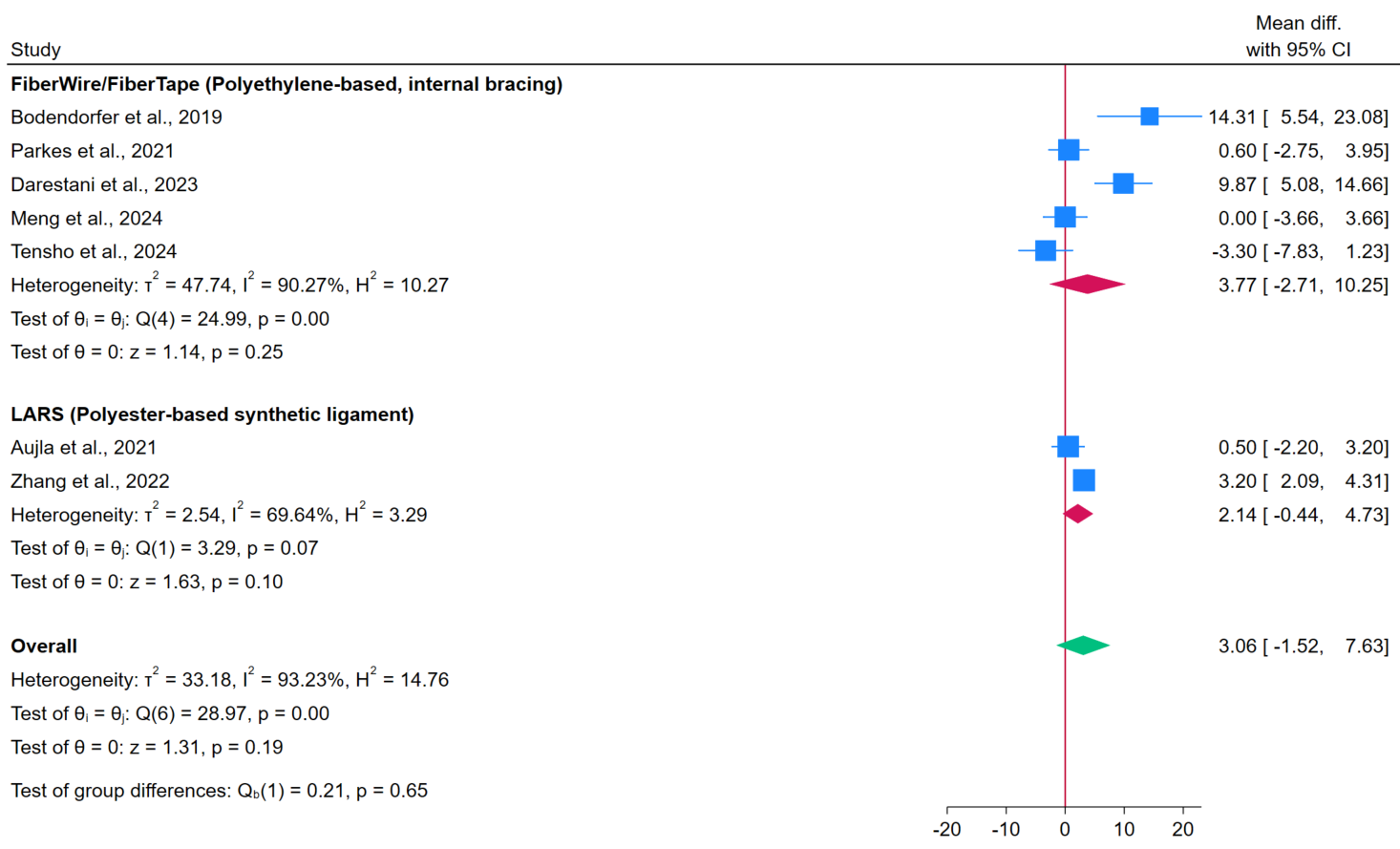


**Figure S12: Forest plot for postoperative IKDC scores; Mid-term follow-up; Subgroup of different materials**. **Effect size reported in mean difference and 95% CI**

**Reference:**

1. Asahina S, Muneta T, Ishibashi T, Yamamoto H (1996) Effects of knee flexion angle at graft fixation on the outcome of anterior cruciate ligament reconstruction. *Arthroscopy* 12(1):70–75. https://doi.org/10.1016/S0749-8063(96)90158-5
2. Aujla RS, Ebert JR, Annear PT (2021) Anterior cruciate ligament reconstruction using autologous hamstrings augmented with the ligament augmentation and reconstruction system versus hamstrings alone: a comparative cohort study. *Orthop J Sports Med* 9(10):23259671211046631. https://doi.org/10.1177/23259671211046631
3. Barrett GR, Field LD (1993) Comparison of patella tendon versus patella tendon/Kennedy ligament augmentation device for anterior cruciate ligament reconstruction: study of results, morbidity, and complications. *Arthroscopy* 9(6):624–632. https://doi.org/10.1016/S0749-8063(93)70019-8
4. Bodendorfer BM, Michaelson EM, Shu HT, Apseloff NA, Spratt JD, Nolton EC, et al (2019) Suture augmented versus standard anterior cruciate ligament reconstruction: a matched comparative analysis. *Arthroscopy* 35(7):2114–2122. https://doi.org/10.1016/j.arthro.2019.02.045
5. Dahlstedt L, Dalén N, Jonsson U (1990) Goretex prosthetic ligament vs. Kennedy ligament augmentation device in anterior cruciate ligament reconstruction: a prospective randomized 3-year follow-up of 41 cases. *Acta Orthop Scand* 61(3):217–224. https://doi.org/10.3109/17453679008993526
6. Daniel AV, Sheth CD, Shubert DJ, Smith PA (2024) Primary anterior cruciate ligament reconstruction with suture tape augmentation: a case series of 252 patients. *J Knee Surg* 37(5):381–390. https://doi.org/10.1055/s-0043-1777993
7. Daniel AV, Smith PA (2024) Less subsequent revision anterior cruciate ligament reconstruction following primary bone–patellar tendon–bone anterior cruciate ligament reconstruction with suture tape augmentation—A retrospective comparative therapeutic trial with 5-year follow-up. *Arthroscopy* 40(9):2455–2464. https://doi.org/10.1016/j.arthro.2024.05.004
8. Daniel AV, Smith PA (2025) Primary all-soft tissue quadriceps tendon autograft anterior cruciate ligament reconstruction with suture tape augmentation resulted in satisfactory patient outcomes and a low graft failure rate in high school and collegiate athletes. *Arthroscopy* 41(1):95–105. https://doi.org/10.1016/j.arthro.2024.06.012
9. Daniel AV, Smith PA (2024) Risk for revision ACLR after primary all-inside quadrupled semitendinosus hamstring tendon autograft ACLR with independent suture tape augmentation: A retrospective cohort study. *Orthop J Sports Med* 12(9):23259671241270308. https://doi.org/10.1177/23259671241270308
10. Daniel AV, Wijdicks CA, Smith PA (2023) Reduced incidence of revision anterior cruciate ligament reconstruction with internal brace augmentation. *Orthop J Sports Med* 11(7):23259671231178026. https://doi.org/10.1177/23259671231178026
11. Duong TD, Tran DT, Do BN, Nguyen TT, Le SM, Le HH (2022) All-inside arthroscopic anterior cruciate ligament reconstruction with internal brace ligament augmentation using semitendinosus tendon autograft: A case series. *Asia Pac J Sports Med Arthrosc Rehabil Technol* 29:15–21. https://doi.org/10.1016/j.asmart.2022.03.001
12. Ebert JR, Annear PT (2019) ACL reconstruction using autologous hamstrings augmented with the ligament augmentation and reconstruction system provides good clinical scores, high levels of satisfaction and return to sport, and a low retear rate at 2 years. *Orthop J Sports Med* 7(10):2325967119879079. https://doi.org/10.1177/2325967119879079
13. Ebert JR, Edwards P, Annear PT (2023) Good clinical scores, no evidence of excessive anterior tibial translation, a high return to sport rate and a low re-injury rate is observed following anterior cruciate ligament reconstruction using autologous hamstrings augmented with suture tape. *Arch Orthop Trauma Surg* 143(8):5207–5220. https://doi.org/10.1007/s00402-023-04805-1
14. Ebert JR, Nairn R, Breidahl W, Annear PT (2022) Double-bundle anterior cruciate ligament reconstruction using autologous hamstrings with LARS augmentation demonstrates comparable outcomes to hamstrings alone, without evidence of synovitis or early osteoarthritis. *Knee Surg Sports Traumatol Arthrosc* 30(7):2320–2328. https://doi.org/10.1007/s00167-022-06949-7
15. Falconer TM, Tusak L, Breidahl WH, Annear PT (2015) The LARS augmented 4-TUNNEL hamstring “hybrid” ACLR graft construction allows accelerated rehabilitation without knee laxity—case series of 111 patients after 2 years. *J Musculoskelet Res* 18(4):1550020. https://doi.org/10.1142/S0218957715500205
16. Garside JC, Bellaire CP, Schaefer EJ, Kim BS, Panish BJ, Elkadi SH, et al (2024) Anterior cruciate ligament reconstruction with suture tape augmentation in the high-risk, young population. *Eur J Orthop Surg Traumatol* 35(1):4. https://doi.org/10.1007/s00590-023-03412-9
17. Grøntvedt T, Engebretsen L, Bredland T (1996) Arthroscopic reconstruction of the anterior cruciate ligament using bone-patellar tendon-bone grafts with and without augmentation. *J Bone Joint Surg Br* 78(5):817–822. https://doi.org/10.1302/0301-620X.78B5.0780817
18. Kdolsky R, Gibbons D, Kwasny O, Schabus R, Plenk Jr H (1997) Braided polypropylene augmentation device in reconstructive surgery of the anterior cruciate ligament: long‐term clinical performance of 594 patients and short‐term arthroscopic results, failure analysis by scanning electron microscopy, and synovial histomorphology. *J Orthop Res* 15(1):1–10. https://doi.org/10.1002/jor.1100150102
19. Kitchen BT, Mitchell BC, Cognetti DJ, Siow MY, Howard R, Carroll AN, et al (2022) Outcomes after hamstring ACL reconstruction with suture tape reinforcement in adolescent athletes. *Orthop J Sports Med* 10(4):23259671221085577. https://doi.org/10.1177/23259671221085577
20. MacDonald PB, Hedden D, Pacin O, Huebert D (1995) Effects of an accelerated rehabilitation program after anterior cruciate ligament reconstruction with combined semitendinosus-gracilis autograft and a ligament augmentation device. *Am J Sports Med* 23(5):588–592. https://doi.org/10.1177/036354659502300512
21. Marcacci M, Zaffagnini S, Neri M, Iacono F, Petitto A, Isola P, et al (1996) Comparative assessment of the results of three ACL reconstruction techniques after a minimum five-year follow-up. J Sports Traumatol Relat Res 18:8–15.
22. **Meng Q, Dai R, Wang C, Shi W, Jiang Y, Liu N, et al** (2024) Additional suture augmentation to anterior cruciate ligament reconstruction with hamstring autografts bring no benefits to clinical results, graft maturation and graft-bone interface healing. BMC Musculoskelet Disord 25(1):301. https://doi.org/10.1186/s12891-024-07051-w
23. **Mohan R, Kwaees TA, Thomas T, Pydisetty R** (2023) Cohort study of short-term outcomes after ACL-reconstruction using autograft with internal brace reinforcement versus isolated autograft demonstrating no significant difference. J Orthop 43:115–120. https://doi.org/10.1016/j.jor.2023.02.010
24. **Moyen B, Jenny J-Y, Mandrino AH, Lerat J** (1992) Comparison of reconstruction of the anterior cruciate ligament with and without a Kennedy ligament-augmentation device: a randomized, prospective study. J Bone Joint Surg Am 74(9):1313–1319. https://doi.org/10.2106/00004623-199274090-00008
25. **Muneta T, Sekiya I, Sakaguchi Y, Yamamoto H, Shinomiya K** (2000) The outcome and complications of ACL reconstruction augmented with a ligament augmentation device for a follow-up period of more than five years. J Long Term Eff Med Implants 10(3):225–238.
26. **Muren O, Dahlstedt L, Dalén N** (2003) Reconstruction of acute anterior cruciate ligament injuries: a prospective, randomised study of 40 patients with 7-year follow-up: No advantage of synthetic augmentation compared to a traditional patellar tendon graft. Arch Orthop Trauma Surg 123:144–147. https://doi.org/10.1007/s00402-003-0497-y
27. **Muren O, Dahlstedt L, Dalén N** (1995) Reconstruction of old anterior cruciate ligament injuries: no difference between the Kennedy LAD-method and traditional patellar tendon graft in a prospective randomized study of 40 patients with 4-year follow-up. Acta Orthop Scand 66(2):118–122. https://doi.org/10.3109/17453679508995512
28. **Nakayama Y, Shirai Y, Narita T, Mori A, Kobayashi K** (2000) Knee functions and a return to sports activity in competitive athletes following anterior cruciate ligament reconstruction. J Nippon Med Sch 67(3):172–176. https://doi.org/10.1272/jnms.67.172
29. **Noyes FR, Barber S** (1992) The effect of a ligament-augmentation device on allograft reconstructions for chronic ruptures of the anterior cruciate ligament. J Bone Joint Surg Am 74(7):960–973. https://doi.org/10.2106/00004623-199274070-00010
30. **Parkes CW, Leland DP, Levy BA, Stuart MJ, Camp CL, Saris DB, et al** (2021) Hamstring autograft anterior cruciate ligament reconstruction using an all-inside technique with and without independent suture tape reinforcement. Arthroscopy 37(2):609–616. https://doi.org/10.1016/j.arthro.2020.10.034
31. **Peterson L, Eklund U, Engström B, Forssblad M, Saartok T, Valentin A** (2014) Long-term results of a randomized study on anterior cruciate ligament reconstruction with or without a synthetic degradable augmentation device to support the autograft. Knee Surg Sports Traumatol Arthrosc 22:2109–2120. https://doi.org/10.1007/s00167-013-2711-5
32. **Roth JH, Kennedy JC, Lockstadt H, McCallum CL, Cunning LA** (1985) Polypropylene braid augmented and nonaugmented intraarticular anterior cruciate ligament reconstruction. Am J Sports Med 13(5):321–336. https://doi.org/10.1177/036354658501300507
33. **Santi MD, Richardson AB** (1994) The ligament augmentation device in hamstring grafts for reconstruction of the anterior cruciate ligament. Am J Sports Med 22(4):524–530. https://doi.org/10.1177/036354659402200414
34. **Saragaglia D, Leroy J, De Sousa B, Tourne Y, Al Zahab MA** (1995) Medium-term results of 173 ligamentoplasties of the anterior cruciate ligament using the MacIntosh technique reinforced by the Kennedy ligament augmentation device (LAD). Knee Surg Sports Traumatol Arthrosc 3:68–74. https://doi.org/10.1007/BF01565463
35. **Sgaglione NA, Del Pizzo W, Fox JM, Friedman MJ, Snyder SJ, Ferkel RD** (1992) Arthroscopic-assisted anterior cruciate ligament reconstruction with the semitendinosus tendon: comparison of results with and without braided polypropylene augmentation. Arthroscopy 8(1):65–77. https://doi.org/10.1016/0749-8063(92)90049-R
36. **Shantanu K, Singh S, Ratha S, Kumar D, Sharma V** (2019) Comparative study of functional outcomes of arthroscopic ACL reconstruction by augmented hamstring graft with fiber tape and hamstring graft alone: A prospective study. Int J Orthop Sci 5(3):165–173. https://doi.org/10.22271/ortho.2019.v5.i3c.1572
37. **Simard SG, Greenfield CJ, Khoury AN** (2024) Anterior cruciate ligament repair with suture tape augmentation of proximal tears and early anterior cruciate ligament reconstruction with suture tape augmentation result in comparable clinical outcomes with anterior cruciate ligament reconstruction at 2-year follow-up. Arthroscopy. https://doi.org/10.1016/j.arthro.2024.02.013
38. **Smith PA, Daniel AV, Stensby JD, Cook CS, Wijdicks CA** (2024) Quadriceps tendon autograft ACL reconstruction with suture tape augmentation: Safe results based on minimum 2-year follow-up MRI. Orthop J Sports Med 12(4):23259671241239275. https://doi.org/10.1177/23259671241239275
39. **Steenbrugge F, Verdonk R, Verstraete K** (2002) Allograft reconstructions for chronic ruptures of the anterior cruciate ligament: augmentation versus non-augmentation. Eur J Orthop Surg Traumatol 12(1):8–13. https://doi.org/10.1007/s005900200002
40. **Struewer J, Ziring E, Ishaque B, Efe T, Schwarting T, Buecking B, et al** (2013) Second-look arthroscopic findings and clinical results after polyethylene terephthalate augmented anterior cruciate ligament reconstruction. Int Orthop 37:327–335. https://doi.org/10.1007/s00264-012-1735-3
41. **Takazawa Y, Ikeda H, Saita Y, Kawasaki T, Ishijima M, Nagayama M, et al** (2017) Return to play of rugby players after anterior cruciate ligament reconstruction using hamstring autograft: return to sports and graft failure according to age. Arthroscopy 33(1):181–189. https://doi.org/10.1016/j.arthro.2016.06.021
42. **Tavakoli Darestani R, Afzal S, Pourmojarab A, Baroutkoub M, Sayyadi S, Barati H** (2023) A comparative analysis of suture-augmented and standard hamstring autograft single-bundle ACL reconstruction outcomes: short-term functional benefits without long-term impact. BMC Musculoskelet Disord 24(1):971. https://doi.org/10.1186/s12891-023-06877-9
43. **Tensho K, Iwaasa T, Koyama S, Shimodaira H, Takahashi T, Takahashi J** (2024) No difference in graft signal intensity on magnetic resonance imaging or clinical outcome between anterior cruciate ligament reconstruction with and without suture augmentation. Arthroscopy. https://doi.org/10.1016/j.arthro.2024.01.017
44. **Thuresson P, Sandberg R, Johansson O, Balkfors B, Westlin N** (1996) Anterior cruciate ligament reconstruction with the patellar tendon‐augmentation or not? A 2‐year follow‐up of 82 patients. Scand J Med Sci Sports 6(4):247–254. https://doi.org/10.1111/j.1600-0838.1996.tb00094.x
45. **von Essen C, Sarakatsianos V, Cristiani R, Stålman A** (2022) Suture tape reinforcement of hamstring tendon graft reduces postoperative knee laxity after primary ACL reconstruction. J Exp Orthop 9(1):20. https://doi.org/10.1186/s40634-022-00465-9
46. **Wilson WT, Kennedy MJ, MacLeod D, Hopper GP, MacKay GM** (2023) Outcomes of anterior cruciate ligament reconstruction with independently tensioned suture tape augmentation at 5-year follow-up. Am J Sports Med 51(14):3658–3664. https://doi.org/10.1177/03635465231198963
47. **Zhang B, Xiang P, Bian S, Wang Y, Wang Y, Ma Y** (2022) Early clinical outcomes of ACL reconstruction using semitendinosus tendon combined with LARS synthetic. Comput Math Methods Med 2022(1):2845114. https://doi.org/10.1155/2022/2845114
