# Supplementary material for: Synthetic augmentation in ACL reconstruction may reduce re‐rupture rates and increase return‐to‐sport rates: A systematic review and meta‐analysis
Source: Knee Surg Sports Traumatol Arthrosc. 2025 Apr 18;34(2):424–35. doi: 10.1002/ksa.12680 (PMC12850593; doi:10.1002/ksa.12680)
Supplement: Supplementary file 2 — Supporting information. [file KSA-34-424-s002.docx]

# **Appendix 1: Literature searches for ACL review**

## Overview

| ***Bibliographic databases and clinical trials registers*** | | |
| --- | --- | --- |
| ***Initial searches*** | | |
| **Database** | **Date searched** | **Number of records** |
| MEDLINE All (via Ovid) | 19/02/24 | 1,040 |
| Embase (via Ovid) | 19/02/24 | 2,181 |
| Science Citation Index (via Web of Science) | 19/02/24 | 1,467 |
| Global Index Medicus (via World Health Organization) | 20/02/24 | 8 |
| Clinicaltrials.gov | 20/02/24 | 29 |
| **Total number of records retrieved: 4,725**  **Duplicates removed (EndNote): 1,532**  **Final number for screening: 3,193** | | |
| ***Updated searches*** | | |
| **Database** | **Date searched** | **Number of records** |
| MEDLINE All (via Ovid) | 17/02/25 | 70 |
| Embase (via Ovid) | 17/02/25 | 223 |
| Science Citation Index (via Web of Science) | 17/02/25 | 134 |
| Global Index Medicus (via World Health Organization) | 17/02/25 | 0 |
| Clinicaltrials.gov | 18/02/25 | 0 |
| **Total number of records retrieved: 427**  **Duplicates removed (EndNote): 65**  **Final number for screening: 362** | | |

**19/02/2024**

**Ovid MEDLINE(R) <1946 to February Week 2 2024>**

1 Anterior Cruciate Ligament/ 12607

2 ((anterior adj2 cruciate$ adj2 ligament$) or ACL).mp. 27606

3 Anterior Cruciate Ligament Reconstruction/ 7297

4 Synthetic augmentation.mp. 50

5 Internal brace.mp. 102

6 Synthetic graft.mp. 684

7 Synthetic ligament.mp. 105

8 tape*.mp. 53204

9 augment*.mp. 197976

10 reinforce*.mp. 107252

11 brace*.mp. or Braces/ 10491

12 knee*.af. 189738

13 exp Anterior Cruciate Ligament Reconstruction/ 7651

14 Anterior Cruciate Ligament Injuries/ 13672

15 1 or 2 or 3 or 13 or 14 27646

16 4 or 5 or 6 or 7 or 8 or 9 or 10 or 11 366699

17 12 and 15 and 16 1188

18 limit 17 to english language 1040

**Embase <1974 to 2024 February 16>**

1 Anterior Cruciate Ligament/ 14104

2 ((anterior adj2 cruciate$ adj2 ligament$) or ACL).mp. 45043

3 Anterior Cruciate Ligament Reconstruction/ 16382

4 Synthetic augmentation.mp. 68

5 Internal brace.mp. 242

6 Synthetic graft.mp. 1396

7 Synthetic ligament.mp. 151

8 tape*.mp. 93852

9 augment*.mp. 296700

10 reinforce*.mp. 158912

11 brace*.mp. or Braces/ 19387

12 knee*.af. 306554

13 exp Anterior Cruciate Ligament Reconstruction/ 16382

14 Anterior Cruciate Ligament Injuries/ 7340

15 1 or 2 or 3 or 13 or 14 45043

16 4 or 5 or 6 or 7 or 8 or 9 or 10 or 11 564811

17 12 and 15 and 16 2363

18 limit 17 to english language 2181

**Web of Science 19/02/2024**

1 "Anterior Cruciate Ligament" (All Fields) or ((anterior ADJ2 cruciate$ ADJ2 ligament$) OR ACL) (All Fields) or "Anterior Cruciate Ligament Reconstruction" (All Fields) or "Anterior Cruciate Ligament Injuries" (All Fields) 48,460
2 "Synthetic augmentation" (All Fields) or "Internal brace" (All Fields) or "Synthetic graft" (All Fields) or "Synthetic ligament" (All Fields) or tape* (All Fields) or augment* (All Fields) or reinforce* (All Fields) or brace* OR Braces (All Fields) 1,104,710

3 knee* (All Fields) [241,127](https://www.webofscience.com/wos/woscc/summary/7bb54316-8bd3-480f-a424-dde85f503ee0-cd1dde5e/relevance/1)

4 #1 AND #2 AND #3 1,541

5 #1 AND #2 AND #3 and English (Languages) 1,467

**Global Index Medicus (via World Health Organization) 20.02.2024**

(tw:(Anterior Cruciate Ligament reconstruction)) OR (tw:(Anterior Cruciate Ligament)) OR (tw:(ACL)) AND (tw:("Synthetic augmentation" OR "Synthetic graft" OR "Internal brace" OR "Synthetic ligament" OR tape OR augment OR reinforce* OR brace* OR Braces**)) AND (tw:(knee*)) 44

Limit to English Language 8

**Clinicaltrials.gov 20.02.2024**

29 trials were retrieved but only four studies have reported their results, the rest are in progress.

**17/02/2025**

**Ovid MEDLINE(R) <1946 to February Week 1 2025>**

1 Anterior Cruciate Ligament/ 12889

2 ((anterior adj2 cruciate$ adj2 ligament$) or ACL).mp. 28979

3 Anterior Cruciate Ligament Reconstruction/ 7983

4 Synthetic augmentation.mp. 53

5 Internal brace.mp. 121

6 Synthetic graft.mp. 717

7 Synthetic ligament.mp. 109

8 tape*.mp. 55163

9 augment*.mp. 209561

10 reinforce*.mp. 112889

11 brace*.mp. or Braces/ 10876

12 knee*.af. 198410

13 exp Anterior Cruciate Ligament Reconstruction/ 8337

14 Anterior Cruciate Ligament Injuries/ 14580

15 1 or 2 or 3 or 13 or 14 29019

16 4 or 5 or 6 or 7 or 8 or 9 or 10 or 11 386087

17 12 and 15 and 16 1253

18 limit 17 to english language 1105

19 limit 18 to yr="2024 -Current" 70

**17/02/2025**

**Embase 1974 to 2025 February 17**

1 Anterior Cruciate Ligament/ 15008

2 ((anterior adj2 cruciate$ adj2 ligament$) or ACL).mp. 47955

3 Anterior Cruciate Ligament Reconstruction/ 18176

4 Synthetic augmentation.mp. 72

5 Internal brace.mp. 284

6 Synthetic graft.mp. 1527

7 Synthetic ligament.mp. 160

8 tape*.mp. 99394

9 augment*.mp. 316565

10 reinforce*.mp. 169312

11 brace*.mp. or Braces/ 20502

12 knee*.af. 323522

13 exp Anterior Cruciate Ligament Reconstruction/ 18176

14 Anterior Cruciate Ligament Injuries/ 9878

15 1 or 2 or 3 or 13 or 14 47955

16 4 or 5 or 6 or 7 or 8 or 9 or 10 or 11 601301

17 12 and 15 and 16 2553

18 limit 17 to english language 2369

19 limit 18 to yr="2024 - Current" 223

**Web of Science 17/02/2025**

1 "Anterior Cruciate Ligament" (All Fields) or ((anterior ADJ2 cruciate$ ADJ2 ligament$) OR ACL) (All Fields) or "Anterior Cruciate Ligament Reconstruction" (All Fields) or "Anterior Cruciate Ligament Injuries" (All Fields) 56,128
2 "Synthetic augmentation" (All Fields) or "Internal brace" (All Fields) or "Synthetic graft" (All Fields) or "Synthetic ligament" (All Fields) or tape* (All Fields) or augment* (All Fields) or reinforce* (All Fields) or brace* OR Braces (All Fields) 1,254,342

3 knee* (All Fields) [262,754](https://www.webofscience.com/wos/woscc/summary/7bb54316-8bd3-480f-a424-dde85f503ee0-cd1dde5e/relevance/1)

4 #1 AND #2 AND #3 1,694

5 #1 AND #2 AND #3 and English (Languages) 1,617

5 #1 AND #2 AND #3 and English (Languages) from 19/02/204 to 17/02/2025 134

**Global Index Medicus (via World Health Organization) 17.02.2025**

(tw:(Anterior Cruciate Ligament reconstruction)) OR (tw:(Anterior Cruciate Ligament)) OR (tw:(ACL)) AND (tw:("Synthetic augmentation" OR "Synthetic graft" OR "Internal brace" OR "Synthetic ligament" OR tape OR augment OR reinforce* OR brace* OR Braces**)) AND (tw:(knee*)) 45

Limit to English Language 8

Limit year from 2024 to 2025 0

**Clinicaltrials.gov 18.02.2025**

No new trial with results were retrieved since 20/02/204.

**Appendix 2: List of excluded studies**

| **Study** | **Reason for exclusion** |
| --- | --- |
| Aichroth PM, Patel DV, Jones CB, Wand JS. A combined intra-and extra-articular reconstruction using a carbon-dacron composite prosthesis for chronic anterior cruciate instability: A two to six-year follow-up study. International orthopaedics. 1991 Sep;15:219-27. | Synthetic prosthesis |
| Allom RJ, Wood JA, Chen DB, MacDessi SJ. The addition of suture tape to the hamstring graft construct does not reduce instrumented knee laxity following ACL reconstruction. Arthroscopy, Sports Medicine, and Rehabilitation. 2022 Apr 1;4(2):e545-51. | Outcome not of interest |
| Ateschrang A, Döbele S, Freude T, Stöckle U, Schröter S, Kraus TM. Acute MCL and ACL injuries: first results of minimal-invasive MCL ligament bracing with combined ACL single-bundle reconstruction. Archives of orthopaedic and trauma surgery. 2016 Sep;136:1265-72. | Augmentation on MCL not ACL |
| Banaś M, Kentel M, Morasiewicz P, Witkowski J, Reichert P. Clinical assessment and comparison of ACL reconstruction using synthetic graft (Neoligaments versus FiberTape). Advances in Clinical and Experimental Medicine. 2021;30(5). | Synthetic graft |
| Bell C, Scholes C, Jegatheesan M, Tuckerman K. The effect of short-graft preparation with tape suspension and screw fixation on loss of knee extension following anterior cruciate ligament reconstruction: A retrospective cross-sectional analysis of public hospital cases from 2015-2017. medRxiv. 2020 Apr 24:2020-04. | Not augmentation techniques. The suture mentioned was to fix the grafts in the tunnels |
| Bettin CC, Throckmorton TW, Miller III RH, Azar FM. Technique for partial transphyseal anterior cruciate ligament reconstruction in skeletally immature athletes: preliminary results. Current Orthopaedic Practice. 2019 Jan 1;30(1):19-25. | Synthetic material not used as augmentation |
| Boss A, Stutz G, Oursin C, Gächter A. Anterior cruciate ligament reconstruction combined with valgus tibial osteotomy (combined procedure). Knee Surgery, Sports Traumatology, Arthroscopy. 1995 Sep;3(3):187-91. | Intervention ineligible (combined procedure) |
| Buda R, Baldassarri M, Perazzo L, Ghinelli D, Faldini C. The biological respect of the posterolateral bundle in ACL partial injuries. Retrospective analysis of 2 different surgical management of ACL partial tear in a population of high-demanding sport patients. European Journal of Orthopaedic Surgery & Traumatology. 2019 Apr 9;29:651-8. | No augmentation |
| Cassard X, Cavaignac E, Maubisson L, Bowen M. Anterior cruciate ligament reconstruction in children with a quadrupled semitendinosus graft: preliminary results with minimum 2 years of follow-up. Journal of Pediatric Orthopaedics. 2014 Jan 1;34(1):70-7. | Not augmentation techniques. The suture mentioned was to fix the grafts in the tunnels |
| Chao TT, Lew WD, Lewis JL, Lindquist C, Hong B, Arendt E. Biomechanical effect of a two‐segment anterior cruciate ligament graft with separate femoral attachments and differing levels of prescribed load sharing. Journal of orthopaedic research. 1992 Nov;10(6):868-77. | Biochemical study |
| Chen CH, Chen WJ, Wu CC, Shih CH. Arthroscopic anterior cruciate ligament reconstruction with quadruple semitendinosus and gracilis tendon autograft: surgical technique and clinical results. Journal of Orthopaedic Surgery. 1998 Dec 1;6(2):61. | Not augmentation techniques. The suture mentioned was to fix the grafts in the tunnels |
| Chen J, Wang C, Xu C, Qiu J, Xu J, Tsai TY, Zhao J. Effects of anterolateral structure augmentation on the in vivo kinematics of ACL-reconstructed knees: Response. The American Journal of Sports Medicine. 2021 Jul;49(9):NP43-4. | Non-synthetic augmentation |
| Dabis J, Wilson A. Repair and augmentation with internal brace in the multiligament injured knee. Clinics in Sports Medicine. 2019 Apr 1;38(2):275-83. | Literature review/commentary paper |
| Dahlstedt L, Dalén N, Jonsson U, Adolphson P. Cruciate ligament prosthesis vs. augmentation: a randomized, prospective 5-year follow-up of 41 cases. Acta Orthopaedica Scandinavica. 1993 Jan 1;64(4):431-3. | Different follow-up of the same study. Excluded to avoid duplication. |
| Drogset JO, Grøntvedt T. Anterior cruciate ligament reconstruction with and without a ligament augmentation device: results at 8-year follow-up. The American journal of sports medicine. 2002 Nov;30(6):851-6. | Different follow-up of the same study. Excluded to avoid duplication. |
| Eggli S, Röder C, Perler G, Henle P. Five year results of the first ten ACL patients treated with dynamic intraligamentary stabilisation. BMC musculoskeletal disorders. 2016 Dec;17:1-5. | ACL repair not reconstruction |
| Elveos MM, Drogset JO, Engebretsen L, Brønn R, Lundemo TO, Gifstad T. Anterior cruciate ligament reconstruction using a Bone–Patellar Tendon–Bone graft with and without a ligament augmentation device: a 25-year follow-up of a prospective randomized controlled trial. Orthopaedic journal of sports medicine. 2018 Nov 16;6(11):2325967118808778. | Different follow-up of the same study. Excluded to avoid duplication. |
| Erden T, Toker B, Toprak A, Taşer Ö. Comparison of the outcomes of isolated anterior cruciate ligament reconstruction and combined anterolateral ligament suture tape augmentation and anterior cruciate ligament reconstruction. Joint diseases and related surgery. 2021 Apr;32(1):129. | Augmentation on ALL not ACL |
| Fules PJ, Goddard RK, Madhav RT, Singh B, Prem H, Jadega HK, Mowbary MA. Instrument-guided semitendinosus-gracilis tendon autograft anterior cruciate ligament replacement using a polyester tape and cortical fixation device. Orthopedics. 2003 Jul 1;26(7):688-92. | Soffix (tape) acts as a fixation device, not an augmentation. |
| Gerasimenko AY, Zhurbina NN, Cherepanova NG, Semak AE, Zar VV, Fedorova YO, Eganova EM, Pavlov AA, Telyshev DV, Selishchev SV, Glukhova OE. Frame coating of single-walled carbon nanotubes in collagen on pet fibers for artificial joint ligaments. International Journal of Molecular Sciences. 2020 Aug 26;21(17):6163. | Method for forming SWCNT/collagen coating for artificial ligaments |
| Gupta PK, Gupta PK, Acharya A, Bansal E. Survivorship and functional outcomes of Anterior Cruciate Ligament Reconstruction with Suture Tape Reinforcement-a prospective study. Journal of Clinical Orthopaedics & Trauma. 2024 Oct 1;57. | Intervention ineligible: deliberately chose small size graft for the intervention group |
| Hamido F, Misfer AK, Al Harran H, Khadrawe TA, Soliman A, Talaat A, Awad A, Khairat S. The use of the LARS artificial ligament to augment a short or undersized ACL hamstrings tendon graft. The Knee. 2011 Dec 1;18(6):373-8. | Intervention ineligible: deliberately chose short or undersized graft |
| Hamido F, Al Harran H, Al Misfer AR, El Khadrawe T, Morsy MG, Talaat A, Elias A, Nagi A. Augmented short undersized hamstring tendon graft with LARS® artificial ligament versus four-strand hamstring tendon in anterior cruciate ligament reconstruction: preliminary results. Orthopaedics & Traumatology: Surgery & Research. 2015 Sep 1;101(5):535-8. | Intervention ineligible: deliberately chose short or undersized graft |
| Harilainen A, Myllynen P. Treatment of fresh tears of the anterior cruciate ligament. A comparison of primary suture and augmentation with carbon fibre. Injury. 1987 Nov 1;18(6):396-400. | ACL repair not reconstruction |
| Ho WP, Lee CH, Huang CH, Chen CH, Chuang TY. Clinical results of hamstring autografts in anterior cruciate ligament reconstruction: a comparison of femoral knot/press-fit fixation and interference screw fixation. Arthroscopy: The Journal of Arthroscopic & Related Surgery. 2014 Jul 1;30(7):823-32. | Not augmentation techniques. The suture mentioned was to fix the grafts in the tunnels |
| Ibrahim SA, Ghafar S, Salah M, Alnas MA, Al Misfer A, Farouk H, Al Haran H, Khirait S. Surgical management of traumatic knee dislocation with posterolateral corner injury. Arthroscopy: The Journal of Arthroscopic & Related Surgery. 2013 Apr 1;29(4):733-41. | Intervention ineligible (combined procedure) |
| Inagaki Y, Kondo E, Kitamura N, Onodera J, Yagi T, Tanaka Y, Yasuda K. Prospective clinical comparisons of semitendinosus versus semitendinosus and gracilis tendon autografts for anatomic double-bundle anterior cruciate ligament reconstruction. Journal of Orthopaedic Science. 2013 Sep;18:754-61. | Graft fashioning techniques, not augmentation |
| Irismetov ME, Usmonov FM, Kholikov AM, Rajabov KN, Tadjinazarov MB. Anterior cruciate ligament reconstruction: Single-bundle hamstring versus double-bundle reconstruction with lavsan (polyethylene terephtalate). European Journal of Molecular and Clinical Medicine. 2020;7(2):951-9. | Full text not available |
| Jonsson H, Elmqvist LG, Karrholm J, Tegner Y. Over-the-top or tunnel reconstruction of the anterior cruciate ligament? A prospective randomised study of 54 patients. The Journal of Bone & Joint Surgery British Volume. 1994 Jan 1;76(1):82-7. | No useful outcome data |
| Kdolsky R, Kwasny O, Schabus R. Synthetic Augmented Repair of Proximal Ruptures of the Anterior Cruciate Ligament: Long-Term Results of 66 Patients. Clinical Orthopaedics and Related Research (1976-2007). 1993 Oct 1;295:183-9. | ACL repair not reconstruction |
| Laupattarakasem W, Mahaisavariya B. Iliotibial band for anterior cruciate ligament reconstruction: A new technique for graft augmentation, placement and fixation. J Med Assoc Thai. 1994 Jul;77:343-50. | Outcome not of interest |
| Lavender C, Singh V, Berdis G, Fravel W, Lamba C, Patel T. Anterior cruciate ligament (ACL) reconstruction augmented with bone marrow concentrate, demineralized bone matrix, autograft bone, and a suture tape (the fertilized ACL). Arthroscopy, Sports Medicine, and Rehabilitation. 2021 Dec 1;3(6):e1719-22. | Intervention ineligible (co-intervention) |
| Lavender CD, Schaver AL, Taylor S, Peluso R, Berdis G, Singh V, Cipriani K, Lycans D, Jasko J, Hewett TE. Anterior Cruciate Ligament Reconstruction Augmentation With Bone Marrow Aspirate Concentrate, Demineralized Bone Matrix, and Suture Tape Shows No Difference in Outcomes—But Faster Functional Recovery—Versus Non-augmented Anterior Cruciate Ligament Reconstruction. Arthroscopy: The Journal of Arthroscopic & Related Surgery. 2024 Jul 22. | Intervention ineligible (co-intervention) |
| Lind M, Feller J, Webster KE. Bone tunnel widening after anterior cruciate ligament reconstruction using EndoButton or EndoButton continuous loop. Arthroscopy: The Journal of Arthroscopic & Related Surgery. 2009 Nov 1;25(11):1275-80. | Fixation, not augmentation |
| Manchado-Herrera I, Motta LM, Blanco G, González J, Garcés GL. Anterior tibial translation and patient-reported outcomes after anterior cruciate ligament reconstruction with a tape locking screw: a 5-year follow-up study. Orthopaedics & Traumatology: Surgery & Research. 2021 Apr 1;107(2):102790. | Fixation, not augmentation |
| Marcacci M, Zaffagnini S, Iacono F, Pia Neri M, Petitto A. Early versus late reconstruction for anterior cruciate ligament rupture: results after five years of followup. The American Journal of Sports Medicine. 1995 Nov;23(6):690-3. | Intervention ineligible / no useful outcome data (a mix of with and without LAD, non-separable) |
| Metso L, Nyrhinen KM, Bister V, Sandelin J, Harilainen A. Comparison of clinical results of anteromedial and transtibial femoral tunnel drilling in ACL reconstruction. BMC Musculoskeletal Disorders. 2020 Dec;21:1-7. | Fixation, not augmentation |
| Metso L, Bister V, Harilainen A, Sandelin J. Anterior cruciate ligament reconstruction with a single hamstring tendon graft and tape locking screw (TLS) fixation leads to good clinical outcome in two years of follow-up. Journal of Orthopaedics. 2024 May 1;51:12-5. | Fixation, not augmentation |
| Minz SD, Choudhary R. Radiological and Functional Outcome of ACL Repair with Internal Brace Augmentation: A Retrospective Study. International Journal of Pharmaceutical and Clinical Research. 2023;15(9):87-92. | The wording of repair/reconstruction not clear |
| Muneta T, Yamamoto H, Ishibashi T, Asahina S, Murakami S, Furuya K. The effects of tibial tunnel placement and roofplasty on reconstructed anterior cruciate ligament knees. Arthroscopy: The Journal of Arthroscopic & Related Surgery. 1995 Feb 1;11(1):57-62. | Outcome not of interest |
| Nakayama Y, Shirai Y, Narita T, Mori A. A follow-up arthroscopy after anterior cruciate ligament reconstruction using the patellar tendon augmented by woven polyester. Nippon Ika Daigaku zasshi. 1997;64(6):512-7. | Outcome not of interest |
| Nakayama Y, Shirai Y, Narita T, Mori A. Enlargement of bone tunnels after anterior cruciate ligament reconstruction. Nippon Ika Daigaku zasshi. 1998;65(5):377-81. | Outcome not of interest |
| Nakayama Y, Shirai Y, Narita T, Mori A. Remodeling of patellar tendon grafts augmented with woven polyester after anterior cruciate ligament reconstruction in humans. Journal of Orthopaedic Science. 1999;4(3):163-70. | Outcome not of interest |
| Nebelung W, Becker R, Merkel M, Ropke M. Bone tunnel enlargement after anterior cruciate ligament reconstruction with semitendinosus tendon using endobutton fixation on the femoral side. ARTHROSCOPY. 1998;14(8):810-5. | Not augmentation techniques. The suture mentioned was to fix the grafts in the tunnels |
| Noyes FR, Mangine RE, Barber S. Early knee motion after open and arthroscopic anterior cruciate ligament reconstruction. American Journal of Sports Medicine. 1987;15(2):149-60. | Interventions are repair, reconstruction with non-synthetic augmentation, and synthetic graft replacement. |
| Orfeuvre B, Pailhe R, Sigwalt L, Duval BR, Lateur G, Plaweski S, Saragaglia D. Anterior cruciate ligament reconstruction with the Tape Locking Screw (TLS) and a short hamstring graft: Clinical evaluation of 61 cases with a minimum 12 months’ follow-up. Orthopaedics & Traumatology: Surgery & Research. 2018 Sep 1;104(5):701-5. | Fixation, not augmentation |
| Orfeuvre B, Pailhé R, Sharma A, Gaillot J, Rubens Duval B, Saragaglia D. Independent clinical appraisal of the Tape Locking Screw (TLS®) anterior cruciate ligament reconstruction technique compared with the hamstring graft technique with a minimum of 12-month follow-up. European Journal of Orthopaedic Surgery & Traumatology. 2019 Aug 1;29:1271-6. | Fixation, not augmentation |
| Ortmaier R, Fink C, Schobersberger W, Kindermann H, Leister I, Runer A. Rückkehr zum Sport nach Ruptur des vorderen Kreuzbandes. Eine Matched-paired-Studie nach Kreuzbandnaht mittels Internal Brace und Ersatzplastik mittels Hamstring-oder Quadrizepssehne (Return to sports after anterior cruciate ligament injury: a matched-pair analysis of repair with internal brace and reconstruction using hamstring or quadriceps tendons). Sportverletz Sportschaden. 2021;35(1):36-44. | Augmentation used in the ACL repair group, not reconstruction group |
| Pacull R, Kalk E, Rongieras F, Bertani A, Gras LL. Anterior cruciate ligament reconstruction with short hamstring grafts: the choice of femoral fixation device matters in controlling overall lengthening. Knee Surgery, Sports Traumatology, Arthroscopy. 2022 Jul;30(7):2215-26. | Fixation, not augmentation |
| Piskopakis A, Totlis T, Achlatis V, Zampeli F, Georgoulis JD, Hantes M, Piskopakis N, Vekris M. Manual and Device-Assisted Hamstring Autograft Tensioning Yield Similar Outcomes following ACL Reconstruction. Journal of Clinical Medicine. 2023 Jul 11;12(14):4623. | Tensioning, not augmentation |
| Plaweski S, Lanternier H. Is quadruple semitendinosus tendon strands autograft a better choice than hamstring autograft for anterior cruciate ligament reconstruction? A comparative study with a mean follow-up of 3 years. European Journal of Orthopaedic Surgery & Traumatology. 2020 Dec;30:1473-9. | Fixation, not augmentation |
| Qiu J, Wang C, Kernkamp WA, Chen J, Xu C, Tsai TY, Zhao J. Augmentation of anterolateral structures of the knee causes undesirable tibiofemoral cartilage contact in double-bundle anterior cruciate ligament reconstruction—a randomized in-vivo biomechanics study. Arthroscopy: The Journal of Arthroscopic & Related Surgery. 2022 Apr 1;38(4):1224-36. | Non-synthetic augmentation |
| Ranger P, Senay A, Gratton GR, Lacelle M, Delisle J. LARS synthetic ligaments for the acute management of 111 acute knee dislocations: effective surgical treatment for most ligaments. Knee Surgery, Sports Traumatology, Arthroscopy. 2018 Dec;26:3673-81. | Intervention ineligible (ACL&PCL) |
| Rao AJ, Macknet DM, Stuhlman CR, Yeatts NC, Trofa DP, Odum SM et al (2021) Allograft augmentation of hamstring autograft in anterior cruciate ligament reconstruction results in equivalent outcomes to autograft alone. Arthroscopy 37:173-182.e172 | Non-synthetic augmentation |
| El Rassi G, Maalouly J, Tawk A, Aouad D. All-inside anterior cruciate ligament reconstruction with augmentation using the native anterior cruciate ligament remnant by suture approximation. Arthroscopy Techniques. 2021 Mar 1;10(3):e647-52. | Surgical technique |
| Rawaf DL, Yasen SK, Borton ZM, Khakha RS, Risebury MJ, Wilson AJ. Anterolateral ligament (ALL) with anterior cruciate ligament (ACL) reconstruction: Rationale, technique & early results. Knee. 2017;24(6):V. | An extraarticular ALL procedure |
| Schenk S, Landsiedl F, Enenkel M. Arthroscopic single-stranded semitendinosus tendon-versus PDS-augmentation of reinserted acute femoral anterior cruciate ligament tears: 7 year follow-up study. Knee Surgery, Sports Traumatology, Arthroscopy. 2006 Apr;14:318-24. | ACL repair not reconstruction |
| Schneider KN, Ahlbäumer G, Gosheger G, Theil C, Weller J, Goth A. Promising functional outcomes following anterior cruciate ligament repair with suture augmentation. Knee Surgery, Sports Traumatology, Arthroscopy. 2023 Jul;31(7):2836-43. | ACL repair not reconstruction |
| Seo SS, Kim CW, Lee CR, Park DH, Kwon YU, Kim OG, Kim CK. Second-look arthroscopic findings and clinical outcomes of meniscal repair with concomitant anterior cruciate ligament reconstruction: comparison of suture and meniscus fixation device. Archives of orthopaedic and trauma surgery. 2020 Mar;140:365-72. | Fixation, not augmentation |
| Severyns M, Plawecki S, Odri GA, Vendeuvre T, Depiesse F, Flez JF, Liguori LA. Correlation of isokinetic testing and ACL failure with the short graft tape suspension technique at six months. Arthroscopy, Sports Medicine, and Rehabilitation. 2022 Apr 1;4(2):e585-90. | Tape suspension technique, not augmentation |
| Shekhar A, Tapasvi S, Williams A. Outcomes of combined lateral meniscus posterior root repair and anterior cruciate ligament reconstruction. Orthopaedic Journal of Sports Medicine. 2022 Mar 11;10(3):23259671221083318. | Suture repair, not augmentation |
| Sinha S, Naik AK, Kumar A, Jacob T, Kar S. Analysis of modified double-bundle anterior cruciate ligament reconstruction with implantless fixation on tibial side. Chinese Journal of Traumatology. 2020 Dec 1;23(06):341-5. | Fixation, not augmentation |
| Steenbrugge F, Verdonk R, Vorlat P, Mortier F, Verstraete K. Repair of chronic ruptures of the anterior cruciate ligament using allograft reconstruction and a ligament augmentation device. Acta orthopaedica belgica. 2001 Jun 1;67(3):252-8. | The comparative version of this study was included. Excluded this to avoid duplication. |
| Strum GM, Larson RL. Clinical experience and early results of carbon fiber augmentation of anterior cruciate reconstruction of the knee. Clinical Orthopaedics and Related Research®. 1985 Jun 1;196:124-38. | Outcome not of interest |
| Szakiel PM, Aksu NE, Kirloskar KM, Gruber MD, Zittel KW, Grieme CV, Geng X, Argintar EH. Rehabilitation and functional outcomes in internally braced and standard ACL reconstructions. Journal of Orthopaedics. 2022 Sep 1;33:95-9. | Outcome not of interest (no useful data) |
| Todhe D, Çipi R, Hysenaj A. Internal Brace Ligament Augmentation versus Anatomical Repair with Hamstrings of the ACL–A Clinical Data Comparison Study. Open Access Macedonian Journal of Medical Sciences. 2022 Dec 10;10(A):1649-52. | ACL repair not reconstruction |
| Toker MB, Erden T, Toprak A, Taşer ÖF. Does anterolateral ligament internal bracing improve the outcomes of anterior cruciate ligament reconstruction in patients with generalized joint hypermobility?. Ulus Travma Acil Cerrahi Derg. 2022 Mar 1;28(3):320-7. | Augmentation on ALL not ACL |
| Uchida R, Shino K, Iuchi R, Tachibana Y, Yokoi H, Nakagawa S, Mae T. Anatomical triple bundle anterior cruciate ligament reconstructions with hamstring tendon autografts: tunnel locations and 2-year clinical outcomes. Arthroscopy: The Journal of Arthroscopic & Related Surgery. 2021 Sep 1;37(9):2891-900. | Suturing, not augmentation |
| Ueki H, Katagiri H, Otabe K, Nakagawa Y, Ohara T, Shioda M, Kohno Y, Hoshino T, Sekiya I, Koga H. Contribution of additional anterolateral structure augmentation to controlling pivot shift in anterior cruciate ligament reconstruction. The American journal of sports medicine. 2019 Jul;47(9):2093-101. | Non-synthetic augmentation |
| Witvoet J, Christel P. Treatment of chronic anterior knee instabilities with combined intra-and extra-articular transfer augmented with carbon-PLA fibers. Clinical Orthopaedics and Related Research®. 1985 Jun 1;196:143-53. | Not clear in design. It seems case-report. |
| Wredmark T, Engström B. Five‐year results of anterior cruciate ligament reconstruction with the Stryker Dacron high‐strength ligament. Knee Surgery, Sports Traumatology, Arthroscopy. 1993 Jun;1(2):71-5. | Intervention eligible / no useful outcome data (Hybrid ACLR & synthetic prothesis, outcome not separable) |
| Yasuda K, Tsujino J, Tanabe Y, Kaneda K. Effects of initial graft tension on clinical outcome after anterior cruciate ligament reconstruction: autogenous doubled hamstring tendons connected in series with polyester tapes. The American journal of sports medicine. 1997 Jan;25(1):99-106. | The polyester tape was not used for augmentation. |
| Ye Z, Xu J, Chen J, Cho E, Cai J, Wu C, Wu X, Li Z, Xie G, Zhao J, Dong S. Effect of anterolateral structure augmentation on graft maturity after anterior cruciate ligament reconstruction: a clinical and MRI follow-up of 2 years. The American Journal of Sports Medicine. 2022 Jun;50(7):1805-14. | Non-synthetic augmentation |

**Appendix 3: Surgical details**

| **Study** | **Graft Type** | **Type of Synthetic Augmentation** | **Method of Femoral Tunnel Drilling** | **Femoral Fixation** | **Tibial Fixation** | **Internal Brace Fixation** |
| --- | --- | --- | --- | --- | --- | --- |
| Asahina et al., 1996 | STG autograft | Ligament augmentation device (LAD) | Not clear | Staple fixation | Not clear | N/A |
| Aujla et al., 2021 | HT (STG) autograft | Ligament Augmentation and Reconstruction System (LARS) | No clear | Suspensory device | Interference screw | N/A |
| Barrett et al., 1993 | BPTB autograft | Ligament augmentation device (LAD) | Not clear | Interference screw | Interference screw | N/A |
| Bodendorfer et al., 2019 | HT autograft or allograft | InternalBrace (FiberTape (Arthrex)) | Transtibial technique | Suspensory device | Suspensory device | SwiveLock® (Arthrex) anchor |
| Dahlstedt et al., 1990 | QT, PT, and prepatellar tissue | Ligament augmentation device (LAD) | Modified over-the-top technique | Screw and washer | Not clear | N/A |
| Daniel et al., 2023 | QT, BTB, or HT autograft | InternalBrace (FiberTape (Arthrex)) | Anteromedial portal technique | Suspensory device | Interference screw | SwiveLock® (Arthrex) anchor |
| Daniel et al., 2024 | QT, BTB, HT autograft; or FGL allograft (LifeNet) | InternalBrace (FiberTape (Arthrex)) | Anteromedial portal technique | QT, HT, and FGL: Suspensory device BTB: Suspensory device | QT, HT, and FGL: Suspensory device BTB: Interference screw | SwiveLock® (Arthrex) anchor |
| Daniel and Smith, 2024 (1) | BPTB autograft | InternalBrace (FiberTape (Arthrex)) | Anteromedial portal technique | Suspensory device | Interference screw | SwiveLock® (Arthrex) anchor |
| Daniel and Smith, 2024 (2) | HT autograft | InternalBrace (FiberTape (Arthrex)) | Anteromedial portal technique | Suspensory device | Suspensory device | SwiveLock® (Arthrex) anchor |
| Daniel and Smith, 2025 | QT autograft | InternalBrace (FiberTape (Arthrex)) | Not clear | Suspensory device | Suspensory device | SwiveLock® (Arthrex) anchor |
| Darestani et al., 2023 | HT autograft | Suture augmentation (FiberWire (Arthrex)) | All-inside technique | Suspensory device | Interference screw | N/A |
| Duong et al., 2022 | STG autograft | InternalBrace (FiberTape (Arthrex)) | All-inside technique | Suspensory device | Suspensory device | SwiveLock® (Arthrex) anchor |
| Ebert et al., 2019 | HT (STG) autograft | Ligament Augmentation and Reconstruction System (LARS) | Anteromedial portal technique | Suspensory device | Interference screw | N/A |
| Ebert et al., 2022 | HT autograft | Ligament Augmentation and Reconstruction System (LARS) | Not clear | Suspensory device | Interference screw | N/A |
| Ebert et al., 2023 | HT autograft | InternalBrace (FiberTape (Arthrex)) | Anteromedial portal technique | Suspensory device | Interference screw | Anchor fixation |
| Falconer et al., 2015 | HT autograft | Ligament Augmentation and Reconstruction System (LARS) | Not clear | Suspensory device | Interference screw | N/A |
| Garside et al., 2024 | BPTB, QA, or HT autograft | InternalBrace (FiberTape (Arthrex)) | Not clear | Not clear | Not clear | SwiveLock® (Arthrex) anchor |
| Grøntvedt et al., 1996 | BPTB autograft | Ligament augmentation device (LAD) | Not clear | Interference screw | Staple fixation | N/A |
| Kdolsky et al., 1997 | BPTB autograft | Ligament augmentation device (LAD) | Over-the-top technique | Screw fixation | Screw fixation | N/A |
| Kitchen et al., 2022 | HT autograft | InternalBrace (FiberTape (Arthrex)) | Outside-in technique | Suspensory device | Interference screw | Not clear |
| Macdonald et al., 1995 | STG autograft | Ligament augmentation device (LAD) | Not clear | Staple | Suture fixation | N/A |
| Marcacci et al., 1996 | PT autograft or fascia lata flap | Ligament augmentation device (LAD) | Not clear | Staple | Staple fixation | N/A |
| Meng et al., 2024 | STG autograft | InternalBrace (FiberTape (Arthrex)) | Anteromedial portal technique | Suspensory device | Interference screw | SwiveLock® (Arthrex) anchor |
| Mohan et al., 2023 | HT autograft | Infinity-Lock neoligament (Xiros) | Anteromedial portal technique | Suspensory device and bone wedge technique | Interference screw | N/A |
| Moyen et al., 1992 | PT autograft | Ligament augmentation device (LAD) | Not clear | Staple | Staple | N/A |
| Muneta et al., 2000 | QTS and STG autograft | Ligament augmentation device (LAD) | Not clear | Not clear | Not clear | N/A |
| Muren et al., 1995 | QT, PT, and prepatellar tissue | Ligament augmentation device (LAD) | Modified over-the-top technique | Screw and washer | Not clear | N/A |
| Muren et al., 2003 | QT, PT, and prepatellar tissue | Ligament augmentation device (LAD) | Modified over-the-top technique | Screw and washer | Not clear | N/A |
| Nakayama et al., 2000 | STG autograft | Woven polyester (Leeds-Keio (Biomet)) | Not clear | Staple | Staple | N/A |
| Noyes et al., 1992 | BLB autograft | Ligament augmentation device (LAD) | Not clear | Interference screw | Interference screw | N/A |
| Parkes et al., 2021 | HT autograft | InternalBrace (FiberTape (Arthrex)) | Anteromedial portal technique | Suspensory device | Suspensory device | SwiveLock® (Arthrex) anchor |
| Peterson et al., 2014 | BTB autograft | Poly(urethane urea) augmentation device (Artimplant AB) | Transtibial technique | Non-resorbable metal screw fixation | Non-resorbable metal screw fixation | N/A |
| Roth et al., 1985 | PT autograft | Ligament augmentation device (LAD) | Over-the-top technique | Not clear | Not clear | N/A |
| Santi et al., 1994 | STG autograft | Ligament augmentation device (LAD) | Not clear | Screw and washer | Not clear | N/A |
| Saragaglia et al., 1995 | QT autograft | Ligament augmentation device (LAD) | Not clear | Not clear | Not clear | N/A |
| Sgaglione et al., 1992 | ST autograft | Ligament augmentation device (LAD) | Not clear | Screw and washer, staple, or suspensory device | Staple | N/A |
| Shantanu et al., 2019 | STG autograft | Fibertape | Not clear | Not clear | Not clear | N/A |
| Simard et al., 2024 | HT autograft | InternalBrace (FiberTape (Arthrex)) | All-inside technique | Button | Not clear | Not clear |
| Smith et al., 2024 | QT autograft | InternalBrace (FiberTape (Arthrex)) | All-inside technique | Suspensory device | Suspensory device | SwiveLock® (Arthrex) anchor |
| Steenbrugge et al., 2002 | Tibialis anterior allograft | Ligament augmentation device (LAD) | Not clear | Staple | Staple | N/A |
| Struewer et al., 2013 | BTB autograft | Polyethylene terephthalate (Trevira) | Transtibial technique | Press-fit positioning in the tunnels and additional joint-distant mini-plate fixation via a lateral incision. | Press-fit fixation and additional joint-distant staple fixation. | N/A |
| Takazawa et al., 2017 | STG autograft | Telos artificial ligaments (Ai-Medic, Tokyo, Japan) | Anteromedial portal technique | Suspensory device | Staple | N/A |
| Tensho et al., 2024 | HT autograft | Suture augmentation (FiberWire (Arthrex)) | Outside-in technique | Suspensory device | A double spike plate (DSP) system (Meira Co., Nagoya, Japan) | N/A |
| Thuresson et al., 1996 | QT autograft | Ligament augmentation device (LAD) | Not clear | Not clear | Not clear | N/A |
| von Essen et al., 2022 | HT or QT autograft | InternalBrace (FiberTape (Arthrex)) | Anteromedial portal technique | Suspensory device | Suspensory device | Button fixation? |
| Wilson et al., 2023 | HT or PT autograft | InternalBrace (FiberTape (Arthrex)) | Transtibial technique | HT: Suspensory device PT: Interference screw | HT: Interference screw PT: Interference screw | SwiveLock® (Arthrex) anchor |
| Zhang et al., 2022 | Semitendinosus tendon autograft | Ligament Augmentation and Reconstruction System (LARS) | Not clear | Suspensory device | Interference screw | N/A |

Note: BTB, bone-patellar tendon-bone; PT, patellar tendon; HT, hamstring tendon; STG, semitendinosus and gracilis tendons; QT, quadriceps tendon; N/A, not applicable.

**Appendix 4: Further results of meta-analysis**


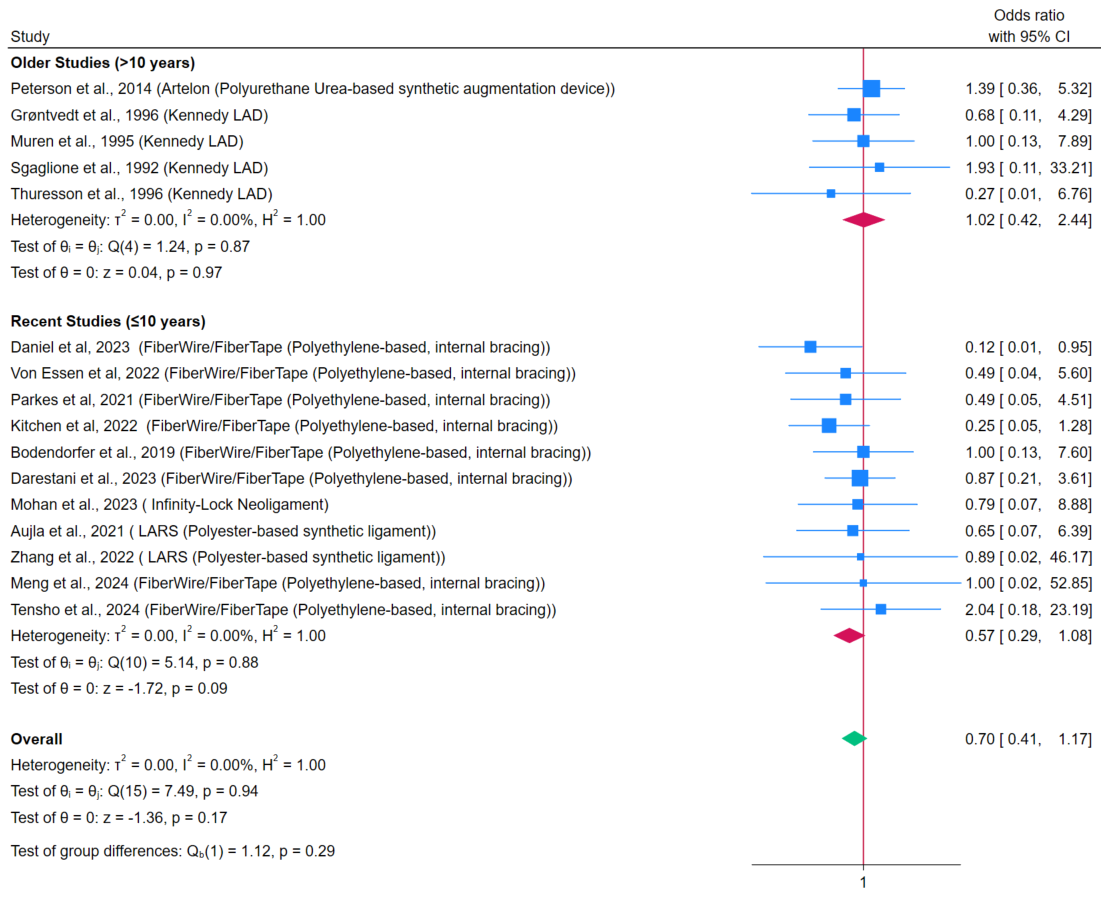


**Figure A1: Forest plot for graft failure rate; Med-term follow-up; Subgroup of publication year**. **Effect size reported in odds ratio and 95% CI**


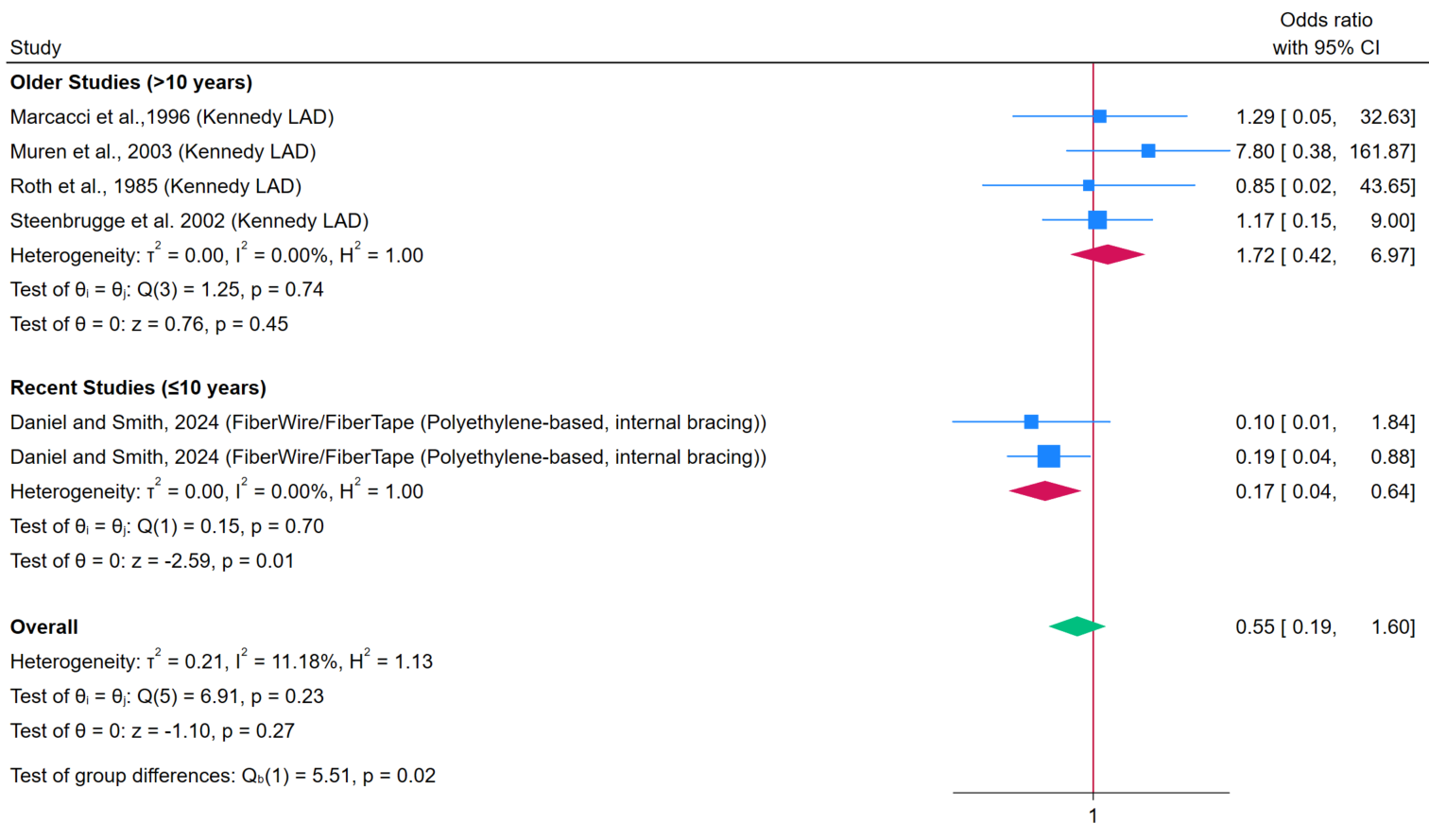


**Figure A2: Forest plot for graft failure rate; Long-term follow-up; Subgroup of publication year. Effect size reported in odds ratio and 95% CI**


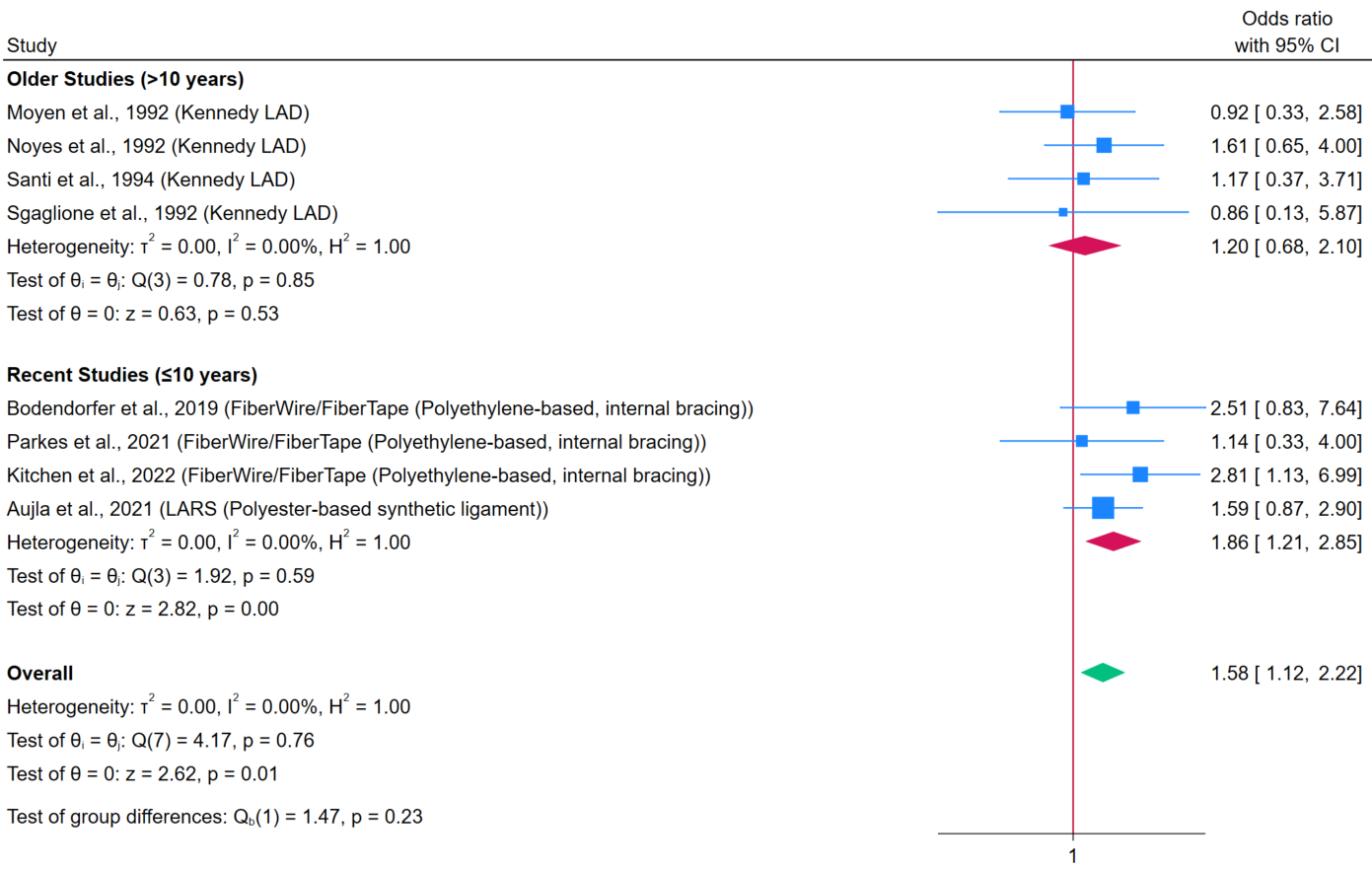


**Figure A3: Forest plot for return to sport rate; Mid-term follow-up; Subgroup of publication year;** **Effect size reported in odds ratio and 95% CI**


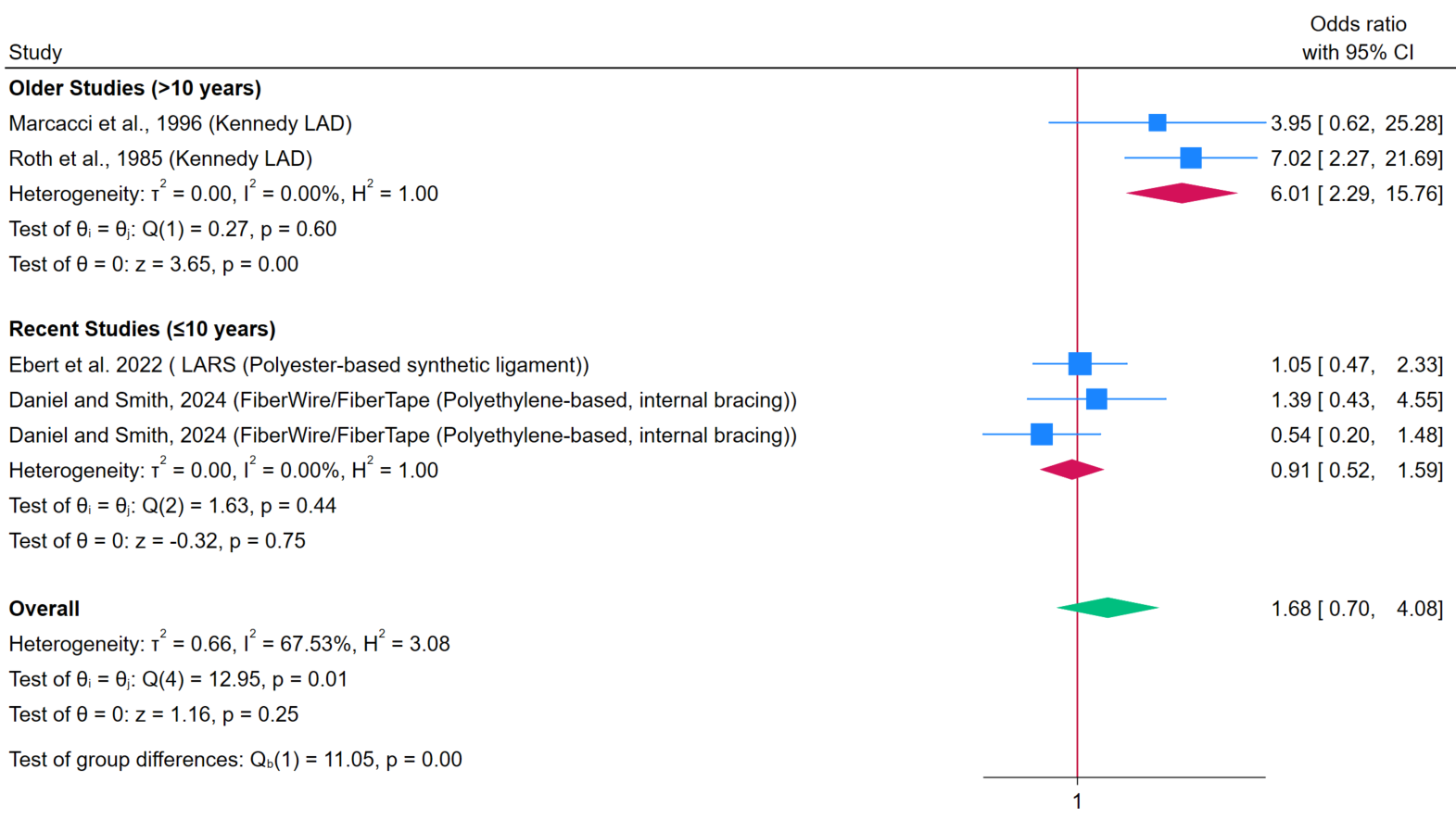


**Figure A4: Forest plot for return to sport rate; Long-term follow-up; Subgroup of publication year**. **Effect size reported in odds ratio and 95% CI.**


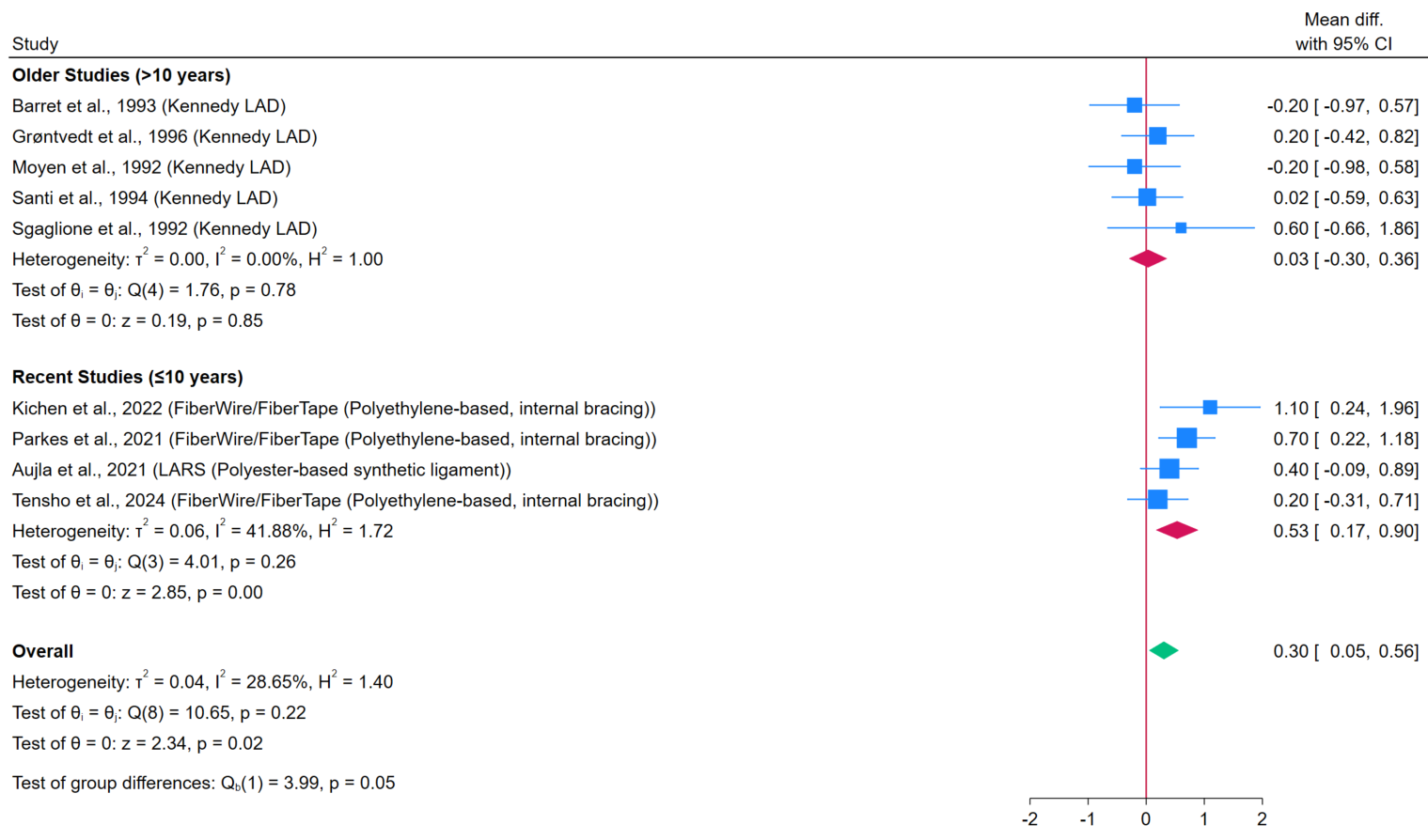


**Figure A5: Forest plot for postoperative Tegner activity score; Mid-term follow-up; Subgroup of publication year.** **Effect size reported in mean difference and 95% CI**


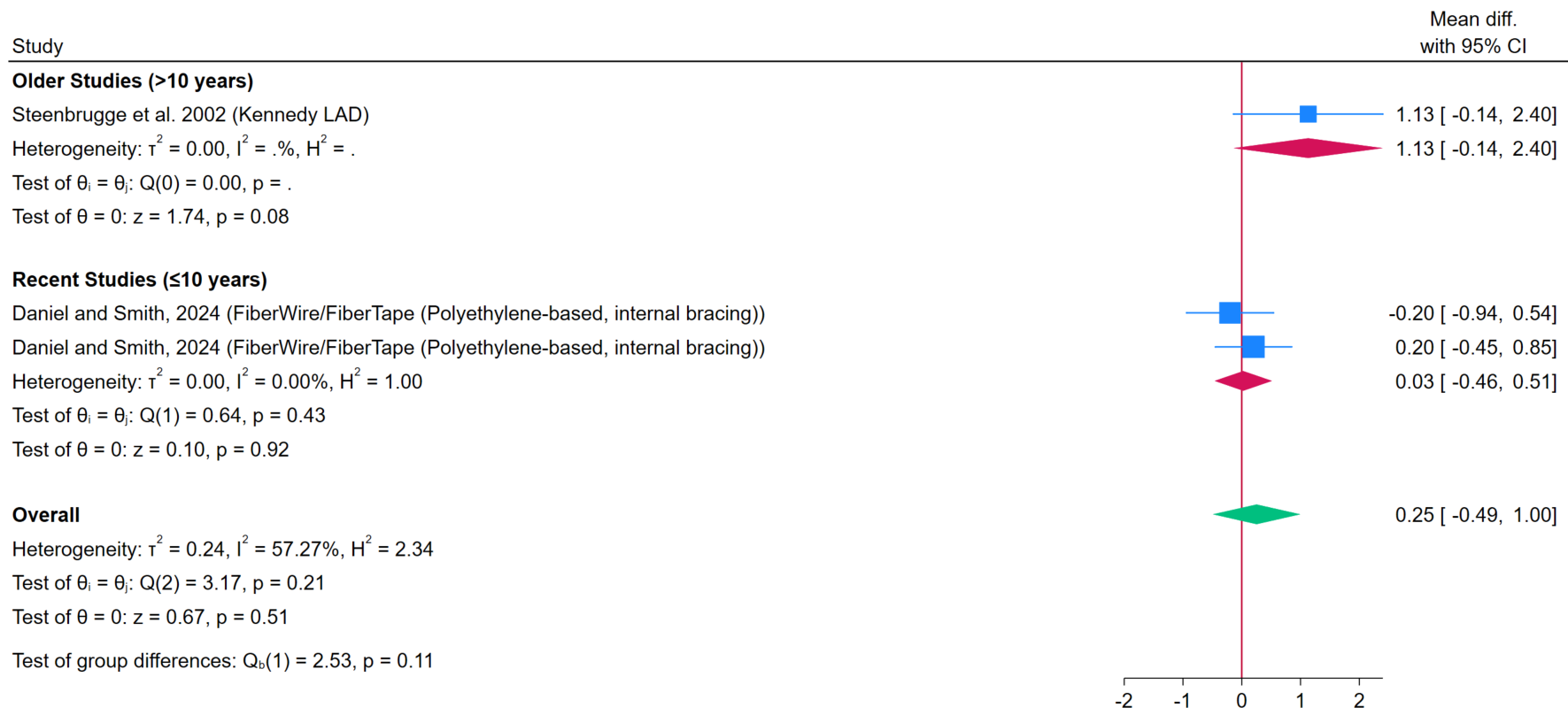


**Figure A6: Forest plot for postoperative Tegner activity score; long-term follow-up; Subgroup of publication year.** **Effect size reported in mean difference and 95% CI**


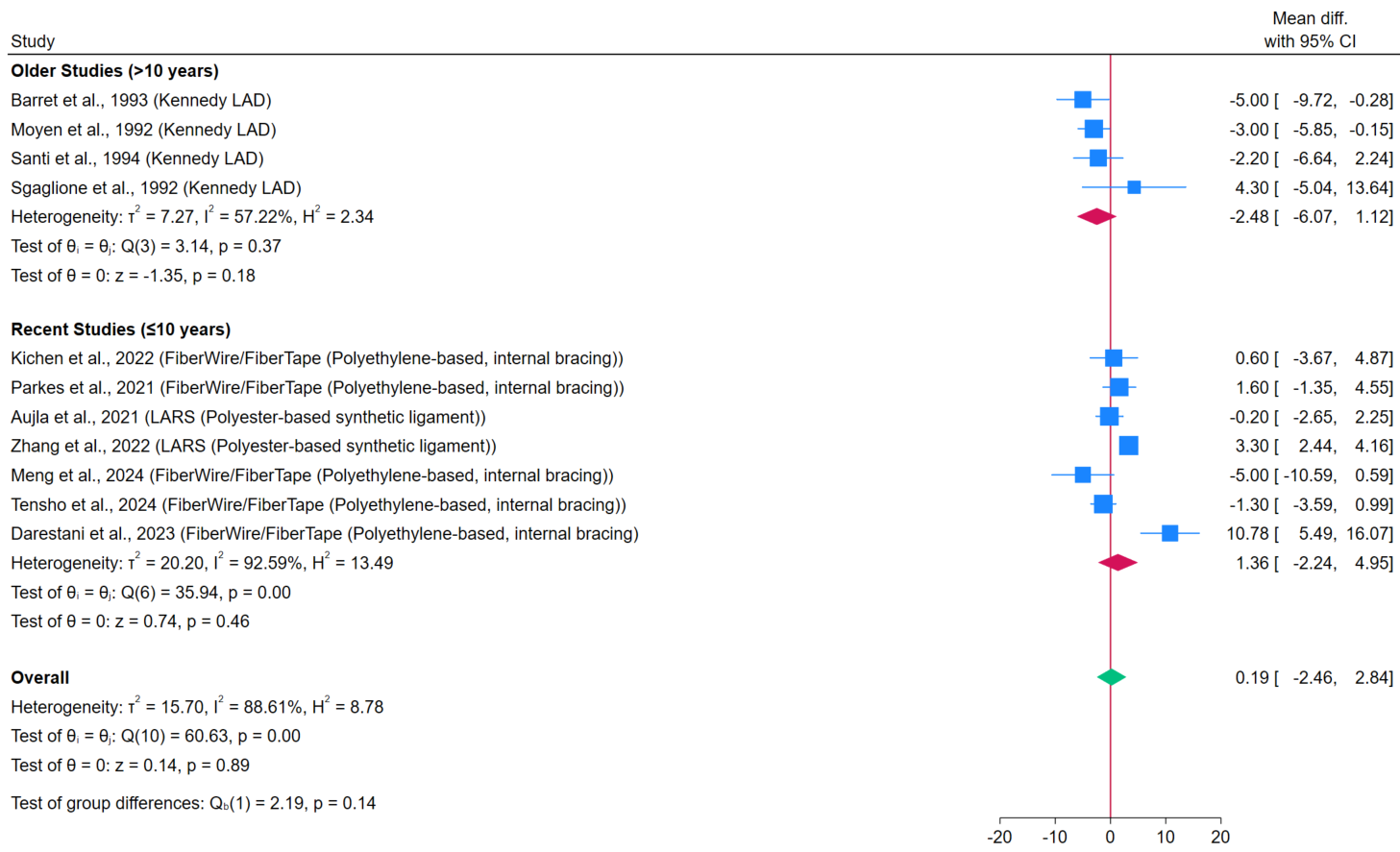


**Figure A7: Forest plot for postoperative Lysholm; Mid-term follow-up; Subgroup of publication year**. **Effect size reported in mean difference and 95% CI**
